# A New Analogue of Echinomycin and a New Cyclic Dipeptide from a Marine-Derived *Streptomyces* sp. LS298

**DOI:** 10.3390/md13116947

**Published:** 2015-11-18

**Authors:** Xin Zhen, Ting Gong, Fu Liu, Pei-Cheng Zhang, Wan-Qi Zhou, Yan Li, Ping Zhu

**Affiliations:** State Key Laboratory of Bioactive Substance and Function of Natural Medicines, Institute of Materia Medica, Chinese Academy of Medical Sciences and Peking Union Medical College, 1 Xian Nong Tan Street, Xicheng District, Beijing 100050, China; E-Mails: zhenxin@imm.ac.cn (X.Z.); gongting@imm.ac.cn (T.G.); liufu@imm.ac.cn (F.L.); pczhang@imm.ac.cn (P.-C.Z.); zhouwanqi@imm.ac.cn (W.-Q.Z.); liyanxiao@imm.ac.cn (Y.L.)

**Keywords:** marine-derived *Streptomyces*, secondary metabolites, antibacterial activity, anti-tumor activity

## Abstract

Quinomycin G (**1**), a new analogue of echinomycin, together with a new cyclic dipeptide, cyclo-(l-Pro-4-OH-l-Leu) (**2**), as well as three known antibiotic compounds tirandamycin A (**3**), tirandamycin B (**4**) and staurosporine (**5**), were isolated from *Streptomyces* sp. LS298 obtained from a marine sponge *Gelliodes carnosa*. The planar and absolute configurations of compounds **1** and **2** were established by MS, NMR spectral data analysis and Marfey’s method. Furthermore, the differences in NMR data of keto-enol tautomers in tirandamycins were discussed for the first time. Antibacterial and anti-tumor activities of compound **1** were measured against 15 drug-sensitive/resistant strains and 12 tumor cell lines*.* Compound **1** exhibited moderate antibacterial activities against *Staphylococcuse pidermidis*, *S. aureus*, *Enterococcus faecium*, and *E. faecalis* with the minimum inhibitory concentration (MIC) values ranged from 16 to 64 μg/mL. Moreover, it displayed remarkable anti-tumor activities; the highest activity was observed against the Jurkat cell line (human T-cell leukemia) with an IC_50_ value of 0.414 μM.

## 1. Introduction

With the emergence of newer resistant forms of infectious diseases and multi-drug resistant (MDR) bacteria and tumors, it has become essential to develop novel and more effective antibiotics [1]. In recent years, numerous studies have discovered that marine-derived actinomycete strains, mainly *Streptomyces* species, have the ability to produce a wide variety of biologically active and structurally unique metabolites. Some of these compounds possess strong antibacterial and anti-tumor activities [2,3,4]. The immense diversity of marine actinomycetes, along with their underutilization, has attracted great attention from researchers to discover novel antibiotics [5,6,7,8].

**Figure 1:**
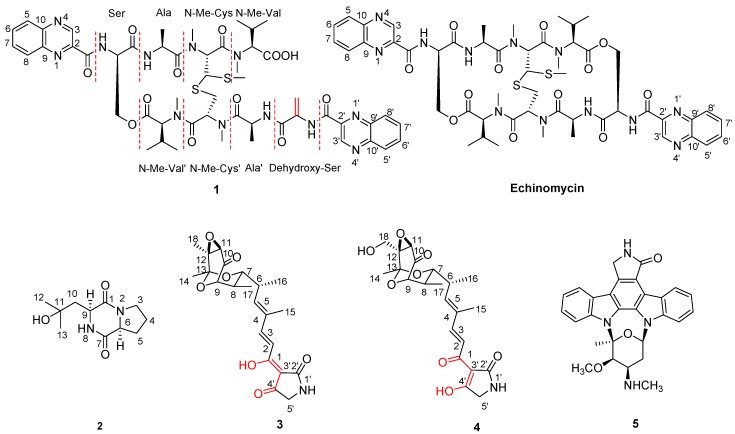
Structures of the isolated compounds **1**–**5** and reference compound echinomycin.

The strain LS298 was obtained from a marine sponge *Gelliodes carnosa* collected from the South China Sea. Based on the 16S rRNA sequence (GenBank accession number FJ937945) analysis [9] and the morphology, this strain was preliminarily identified as *Streptomyces* sp. Our previous studies have shown that the secondary metabolites of this strain contain echinomycin, cyclic dipeptides, and esters [10]. Among these compounds, echinomycin, a bifunctional DNA intercalator, is the predominantly and biologically active constituent against the Gram-positive and Gram-negative bacteria and also shows good anti-tumor activity [11,12,13,14]. Our continued search for echinomycin analogues or other novel antibiotics from extracts of large scale fermentation led to the isolation of two new compounds quinomycin G (**1**) and cyclo-(l-Pro-4-OH-l-Leu) (**2**), as well as three known compounds tirandamycin A (**3**), tirandamycin B (**4**), and staurosporine (**5**) (Figure 1). Structurally, quinomycin G (**1**) possessed a terminal double bond in one of the Ser groups. Cyclo-(l-Pro-4-OH-l-Leu) (**2**) was a new cyclic dipeptide. Tirandamycin A (**3**) was the 1-enol-4′-keto form, while tirandamycin B (**4**) was 1-keto-4′-enol form. It is the first time to reveal this form of tirandamycin B explicitly. In addition, antibacterial and anti-tumor activities of compound **1** were evaluated against 15 drug-resistant/sensitive strains and 12 tumor cell lines.

## 2. Results and Discussion

### 2.1. Structure Elucidation of Compounds **1**–**5**

**Figure 2:**
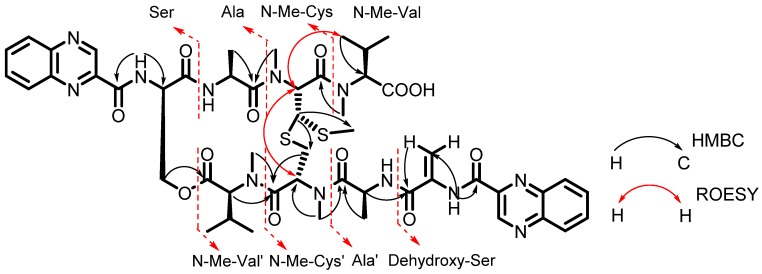
Key HMBC and ROESY correlations of compound **1**.

Quinomycin G (**1**) was obtained as an amorphous yellow powder, a molecular formula of C_51_H_64_N_12_O_12_S_2_ was determined by HRESIMS (*m*/*z* 1101.4288 [M + H]^+^, calcd for C_51_H_65_N_12_O_12_S_2_, 1101.4286), requiring 26 degrees of unsaturation. The chemical structure of **1** was adumbrated as an echinomycin analogue by the close similarity of its molecular formula and ultraviolet spectral properties (λ_max_ (log ε) 245.2 nm (2.6), 325.8 nm (1.9), respectively) to those of echinomycin [10]. The ^1^H NMR spectrum of **1** (Table 1) displayed four NH resonances (δ_H_: 10.67 (1H, s), 9.20 (1H, d, *J* = 9.5 Hz), 9.01 (1H, d, *J* = 9.5 Hz), 7.83 (1H, overlap)); 12 aromatic protons signals (δ_H_: 9.68 (1H, s), 9.63 (1H, s), 8.27 (1H, d, *J* = 8.0 Hz), 8.20 (3H, d, *J* = 8.0 Hz), 7.95–7.97 (2H, overlap), 7.84–7.89 (2H, overlap), 6.90 (1H, brs), 6.11 (1H, brs)); two methylene resonances (δ_H_: 5.02 (1H, dd, *J* = 11.5, 3.0 Hz), 4.67 (1H, d, *J* = 11.5 Hz); 3.47 (1H, dd, *J* = 16.0, 5.0 Hz), 2.54 (1H, d, *J* = 16.0 Hz)); ten methine signals (δ_H_: 6.03 (1H, d, *J* = 4.0 Hz), 5.70 (1H, s), 5.37 (1H, d, *J* = 9.0 Hz), 5.28 (1H, m), 4.85 (1H, m), 4.47 (1H, d, *J* = 11.0 Hz), 3.74 (1H, d, *J* = 2.0 Hz), 3.42 (1H, d, *J* = 10.5 Hz), 2.49 (1H, m), 2.27 (1H, m)); 11 methyl signals in the upfield region, including four N-Me groups (δ_H_: 3.37 (3H, s), 3.15 (3H, s), 3.02 (3H, s), 2.97 (3H, s)), one S-Me group (δ_H_: 2.07 (3H, s)). 51 carbons were observed in the ^13^C NMR spectrum of compound **1** (Table 1), including ten ester/amide carbonyls (δ_C_: 172.2 (2C), 171.5, 169.9 (2C), 169.8, 168.3, 163.6, 163.2, 161.9) and 18 sp^2^ carbon signals (δ_C_: 143.9 (2C), 143.7, 143.5, 143.4, 142.4, 140.3 (2C), 133.0, 132.4, 131.9, 131.6, 131.0, 130.0 (2C), 129.4 (2C), 104.3). Comprehensive analysis of the ^1^H-^1^H COSY (Supplementary Materials Figure S8) and HSQC of compound **1**, indicated that compound **1** was comprised of two quinoxalines and eight amino acid moieties (two N-Me-Val, two Ala, two N-Me-Cys, one Ser, and one Dehydroxy-Ser) (Figure 2). The connections between amino acids moieties were confirmed by an HMBC experiment. The HMBCs ((H-α (δ_H_: 4.85 (1H, m) of Ala′ to the C=O (δ_C_: 163.2) of Dehydroxy-Ser; N-CH_3_ (δ_H_: 3.37 (3H, s) of N-Me-Cys′ to the C=O (δ_C_: 172.2) of Ala′; N-CH_3_ (δ_H_: 3.02 (3H, s) of N-Me-Val′ to the C=O (δ_C_: 171.5) of N-Me-Cys′; H-β (δ_H_: 4.67 (1H, d, *J* = 11.5 Hz) of Ser to the C=O (δ_C_: 169.9) of N-Me-Val′; NH (δ_H_: 9.20 (1H, d, *J* = 9.5 Hz) of Ala to the C=O (δ_C_: 169.9) of Ser; N-CH_3_ (δ_H_: 3.15 (3H, s) of N-Me-Cys to the C=O (δ_C_: 172.2) of Ala; N-CH_3_ (δ_H_: 2.97 (3H, s) of N-Me-Val to C=O (δ_C_: 168.3) of N-Me-Cys)), indicated that the connections were Dehydroxy-Ser-Ala′-N-Me-Cys′-N-Me-Val′-Ser-Ala-N-Me-Cys-N-Me-Val. Above all evidence, compound **1** was structurally similar to echinomycin. The only difference between them was the presence of a double bond (δ_H_: 6.90 (1H, brs), 6.11 (1H, brs); δ_C_: (133.0, 104.3)) in compound **1**. In the HMBC spectrum, the methylene protons (δ_H_: 6.90 (1H, brs), 6.11 (1H, brs)) to C=O (δ_C_: 163.2), confirmed that the double bond originated from the Ser. On the basis of the above information, all protons and carbon resonances were assigned and the planar structure of compound **1** was established. Because the planar differences in the structures of compound **1** and echinomycin cause the changes on spatial configurations, the NMR spectral data, especially ^1^H NMR spectral data of compound **1** were different with that of echinomycin. The appearance of double bond of Dehydroxy-Ser may make the quinoxaline, amide, alkene, and carbonyl groups form a large conjugate plane (Supplementary Materials Figure S9). The CH_3_ of the Ala′ positioned in the shielding area, so its ^1^H NMR spectral data upfielded to δ_H_: 0.19.

**Table 1:** ^1^H (500 MHz) and ^13^C (125 MHz) NMR Date for Compound **1** (CDCl_3_).

Position	δ_H_, mult (*J* in Hz)	δ_C_	Position	δ_H_, mult (*J* in Hz)	δ_C_	Position	δ_H_, mult (*J* in Hz)	δ_C_
Quinoxaline			β	5.02, dd (11.5, 3.0)	65.8	α	5.28, m	45.6
2		143.9		4.67, d (11.5)		β	1.35, d (6.0)	18.3
2′		143.9	C=O		169.9	C=O		172.2
3	9.68, s	143.5	Dehydroxy-Ser			Ala′		
3′	9.63, s	143.7	NH	10.67, s		NH	7.83 ^a^	
5	8.20, d (8.0)	129.4	α		133.0	α	4.85, m	44.9
5′	8.20, d (8.0)	129.4	β	6.90, brs; 6.11, brs	104.3	β	0.19, d (6.0)	16.4
6	7.84–7.89 ^a^	132.4	C=O		163.2	C=O		172.2
6′	7.84–7.89 ^a^	131.9	N-Me-Cys			N-Me-Val		
7	7.95–7.97 ^a^	131.6	N-Me	3.15, s	33.2	N-Me	2.97, s	29.0
7′	7.95–7.97 ^a^	131.0	α	5.70, s	60.4	α	3.42, d (10.5)	65.8
8	8.27, d (8.0)	130.0	β	3.74, d (2.0)	52.1	β	2.27, m	27.4
8′	8.20, d (8.0) ^a^	130.0	S-Me	2.07,s	13.4	γ	1.04 ^a^	19.5
9		140.3	C=O		168.3	γ	0.97, d (6.0)	18.5
9′		140.3	N-Me-Cys′			COOH		169.8
10		143.4	N-Me	3.37, s	33.8	N-Me-Val′		
10′		142.4	α	6.03, d (4.0)	61.1	N-Me	3.02, s	28.8
C=O		163.6	β	3.47, dd (16.0, 5.0)	29.2	α	4.47, d (11.0)	64.6
C=O′		161.9		2.54, d (16.0)		β	2.49, m	28.6
Ser			C=O		171.5	γ	1.06, d (7.0)	19.7
NH	9.01, d (9.5)		Ala			γ	1.03, d (7.0)	18.8
α	5.37, d (9.0)	52.6	NH	9.20, d (9.5)		C=O		169.9

^a^ Overlapping signals.

Marfey’s method was employed to assign the absolute configurations of the amino acid residues resulting from acid hydrolysis of **1** [15,16]. The 1-fluoro-2,4-dinitrophenyl-5-l-alanine amide (FDAA) derivatives of the acid hydrolysate of **1** and the authentic d- and l-amino acids were subjected to HPLC analysis. The absolute configurations of all amino acid residues in **1** except for N-Me-Cys were established by comparing their HPLC retention times with those of the corresponding authentic d- and l- amino acid standards (Table 2, Supplementary Materials Figure S17). The ROESY correlation between H-α of N-Me-Cys (δ_H_: 5.70) and H-α of N-Me-Cys′ (δ_H_: 6.03) showed they necessarily positioned *cis* to each other. In other words, these two N-Me-Cys were either d or l amino acids. The absolute configuration of the N-Me-l-Cys was confirmed by the ROESY correlation between H-α of N-Me-Cys (δ_H_: 5.70) and H-γ of N-Me-l-Val (δ_H_: 0.97) (Figure 2), because this correlation was observed implying H-α of N-Me-Cys (δ_H_: 5.70) located at β-orientaion as same as H-γ of N-Me-l-Val (δ_H_: 0.97). The results indicated that the amino acid residues were l-Ala/Ala′, d-Ser, N-Me-l-Val/N-Me-l-Val′ and N-Me-l-Cys/N-Me-l-Cys′ configurations.

**Table 2:** The retention times of 1-fluoro-2,4-dinitrophenyl-5-l-alanine amide (FDAA) derivatives of the hydrolysates of **1**–**2** and amino acids standards.

	Retention Time (T_R_) Min		
	Amino Acid Standards	Compound 1	Compound 2
l-Ser	15.787	-	-
d-Ser	16.422	16.649	-
l-Ala	22.125	21.952	-
d-Ala	23.789	-	-
N-Me-l-Val	17.499	17.528	-
N-Me-d-Val	19.732	-	-
l-Pro	14.544	-	14.677
d-Pro	17.528	-	-
FDAA	19.772	19.804	19.782

Thus, as shown in **1** (Figure 1), the absolute stereochemistry of this novel echinomycin analogue was assigned and it was given the trivial name quinomycin G.

**Figure 3:**
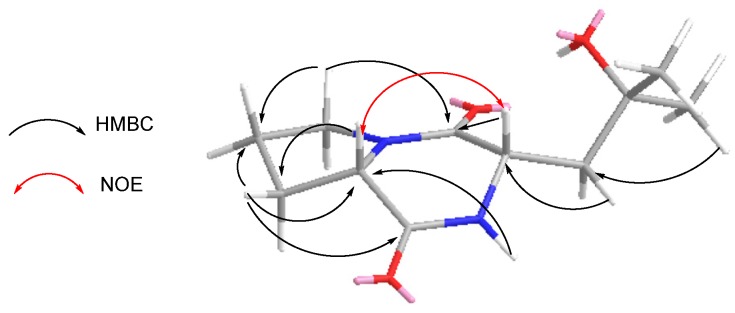
The Key HMBC and NOE correlations of compound **2**.

Cyclo(l-Pro-4-OH-l-Leu) (**2**) was isolated as a white powder, its molecular formula C_11_H_18_N_2_O_3_ was established upon the analysis of the HRESIMS peak at *m*/*z* 249.1209 [M + Na]^+^, indicating four degrees of unsaturation. The ^1^H NMR spectrum of **2** (Table 3) revealed one NH resonance (δ_H_: 7.66 (1H, s)); two methine signals (δ_H_: 4.23 (1H, d, *J* = 10.5 Hz), 4.08 (1H, t, *J* = 8.0 Hz)); four methylene resonances (δ_H_: 3.56 (2H, m); 2.01 (1H, m), 1.89 (1H, m); 2.36 (1H, dd, *J* = 8.5, 2.5 Hz), 2.10 (1H, m); 2.32 (1H, dd, *J* = 14.5, 2.0 Hz), 1.79 (1H, dd, *J* = 14.5, 11.0 Hz)); two methyl signals in the upfield region (δ_H_: 1.31 (1H, s), 1.35 (1H, s)). The ^13^C NMR spectra of **2** (Table 3) showed a total of 11 carbon resonances, and they were classified as two amide carbonyls (δ_C_: 169.7, 166.1), three methines (δ_C_: 70.9, 58.8, 53.2), four methylene signals (δ_C_: 45.6, 41.1, 28.3, 22.6), two methyl signals (δ_C_: 32.3, 27.4). Further analysis of the NMR data confirmed the existence of moieties of Pro and 4-hydroxyl-Leu. In the HMBC experiment (Figure 3), the correlations of H-3 (δ_H_: 3.56 (2H, m)), and H-9 (δ_H_: 4.23 (1H, d, *J* = 10.5 Hz)) with C-1 (δ_C_: 166.1); H-5 (δ_H_: 2.36 (1H, dd, *J* = 8.5, 2.5 Hz), 2.10 (1H, m)) and H-8 (δ_H_: 7.66 (1H, s)) with C-6 (δ_C_: 58.8) confirmed the planar structure for compound **2** was cyclo(Pro-4-hydroxyl-Leu). The relative stereochemistry of **2** was deduced from the NOE spectrum (Figure 3). When irradiating H-6 at δ_H_: 4.08 and H-9 at δ_H_: 4.23, the integration values of H-9 and H-6 were enhanced respectively, which showed that H-6 and H-9 positioned *cis* to each other. That is to say relative configurations of Pro and 4-hydroxyl-Leu were either l or d configurations. On the basis of Marfey’s method, the presence of l-Pro in **2** compared with the appropriate amino acid standards (Table 2, Supplementary Materials Figure S17), determined the configuration of compound **2** as cyclo(l-Pro-4-hydroxyl-l-Leu).

**Table 3:** ^1^H (500 MHz) and ^13^C (125 MHz) NMR Date for compound **2** (CDCl_3_).

No.	δ_H_, mult (*J* in Hz)	δ_C_	No.	δ_H_, mult (*J* in Hz)	δ_C_
1		166.1	7		169.7
2			8	7.66, s	
3	3.56, m	45.6	9	4.23, d (10.5)	53.2
4α	1.89, m	22.6	10α	2.32, dd (14.5, 2.0)	41.1
4β	2.01, m		10β	1.79, dd (14.5, 11.0)	
5α	2.36, dd (8.5, 2.5)	28.3	11		70.9
5β	2.10, m		12	1.31, s	27.4
6	4.08, t (8.0)	58.8	13	1.35, s	32.3

In our ongoing investigation of active metabolites, tirandamycins A (**3**) [17] and B (**4**) [18] were obtained and their structures were elucidated by the MS, ^1^H, ^13^C NMR, HSQC, HMBC spectral analysis (Supplementary Materials Figure S27) and comparison of the respective spectral data with those found in the literature. Surprisingly, their ^1^H and ^13^C NMR spectral data showed significant differences at positions 1, 2, 3 and 5 (Table 4), though they were tested in the same solvent (DMSO-*d*_6_). Subsequently, this inspired us to study the structural distinction between them. The literature survey indicated that the substituent groups on bicyclic ketal moiety have little influence on the NMR spectral data of the long conjugated system [17,18,19,20,21,22]. Therefore, we proposed that the distinct differences of NMR spectral data were caused by the positions of the enolic hydroxy and carbonyl group. Compared with C-1 at δ_C_: 173.5 in tirandamycin A (**3**), the data of C-1 in tirandamycin B (**4**) moved to the downfield at δ_C_: 181.0, implying that **4** should be in 1-keto-4′-enol form. The keto-enol tautomer existed widely in the structure of natural products, and the rules of NMR data of these two tautomers have been studied [23], which also supported that tirandamycin B (**4**) was the 1-keto-4′-enol form. It is the first time to reveal the 1-keto-4′-enol form of tirandamycin B explicitly. Because the structures of 1-keto-4′-enol form of tirandamycins were unclear previously, the assignments of the NMR data of these compounds were not correct [21,22]. Herein, we summarized the trend in NMR data of keto-enol tautomers exist in tirandamycins in order to raise concern on the structural and NMR data differences between these two forms. In the ^13^C NMR spectrum, when the structure was in 1-enol-4′-keto form just like tirandamycin A, the carbon signals occurred at approximately δ_C_: 173.5 (C-1), 116.2 (C-2), 147.9 (C-3) and 143.7 (C-5), however the carbons of the 1-keto-4′-enol form as tirandamycin B resonated at approximately δ_C_: 181.0 (C-1), 124.8 (C-2), 143.2 (C-3) and 137.9 (C-5). More importantly, three olefinic protons had obvious differences in these two tautomers in the ^1^H NMR spectrum, δ_H_: 7.05 (H-2) 7.47 (H-3) and 6.19 (H-5) in 1-enol-4′-keto form changed to δ_H_: 7.55 (H-2) 7.14 (H-3) and 5.81 (H-5) in 1-keto-4′-enol form. The chemical shift of H-2 at δ_H_: 7.55 increased abnormally, which may be due to the shielding effect of the double bond (1C=O). According to the above results and literature survey [17,18,19,20,21,22,24], we also summed up a brief rule that if the ^1^H NMR data of H-5 is more than δ_H_: 6.00, the structure of tirandamycin is in 1-enol-4′-keto form, otherwise, it is in the other form.

**Table 4:** The ^1^H (500 MHz) and ^13^C (125 MHz) NMR Date for Tirandamycins A and B (DMSO-*d*_6_).

No.	Tirandamycin A		Tirandamycin B	
	δ_H_, mult (*J* in Hz)	δ_C_	δ_H_, mult (*J* in Hz)	δ_C_
1		173.5		181.0
2	7.05, d (15.5)	116.2	7.55, d (15.5)	124.8
3	7.47, d (15.5)	147.9	7.14, d (15.5)	143.2
4		134.1		134.2
5	6.19, d (9.5)	143.7	5.81, d (10.0)	137.9
6	2.88, m	33.9	2.83, m	33.5
7	3.77, dd (9.0, 2.0)	75.8	3.73, m	76.1
8	1.81, m	33.9	1.77, m	33.7
9	4.03, d (6.5)	77.8	4.04, d (6.5)	77.7
10		202.8		202.5
11	3.47, s	59.9	3.56, s	56.0
12		56.7		59.8
13		96.2		95.3
14	1.44, s	22.4	1.43, s	23.3
15	1.84, s	12.0	1.79, s	12.5
16	1.07, d (7.0)	16.6	1.05, d (6.5)	16.9
17	0.62, d (7.0)	11.0	0.62, d (6.5)	11.1
18	1.40, s	15.1	3.84, dd (12.5, 3.5) 3.74, dd (12.5, 3.5)	56.5
1′	8.74, s		7.58, s	
2′		176.0		177.9
3′		100.7		101.2
4′		193.9		192.9
5′	3.77, dd (9.0, 2.0) 3.75, dd (9.0, 2.0)	51.4	3.47, s	50.2
18-OH			5.02 brs	

The variable temperature experiments for ^1^H NMR of tirandamycins A (**3**) and B (**4**) were employed for studying the tautomerizm of keto-enol tirandamycins and the test temperatures were set at 40, 60, 80 °C. With the increase of test temperature, the structure of tirandamycin A (**3**) was still in the 1-enol-4′-keto form (Supplementary Materials Figure S21), but the structure of tirandamycin B (**4**) gradually transformed to 1-enol-4′-keto form (Supplementary Materials Figure S26). The results suggested that in the DMSO-*d*_6_ solution, tirandamycin A (**3**) was stable in 1-enol-4′-keto form, while tirandamycin B (**4**) was more stable in 1-keto-4′-enol form than the other form. The reason may be the structure itself or external factors, which need to be further investigated.

The known antibiotic staurosporine (**5**) was characterized by comparison of the respective spectral data (MS, ^1^H, ^13^C NMR) with those found in the literature [25].

### 2.2. Biological Assays

The novel echinomycin analogue compound **1** was assayed for antibacterial activities against *Staphylococcuse pidermidis* (ATCC 12228 (MSSE), 12-6(MSSE), 12-8(MRSE)), *Staphylococcus aureus* (ATCC 29213(MSSA), ATCC 33591(MRSA), 15(MSSA), 12-28(MSSA), 12-33(MRSA)), *Enterococcus faecium* (ATCC 700221(VRE), 12-1(VRE), 12-3(VSE)), and *Enterococcus faecalis* (ATCC 292121(VSE), ATCC 51299 (VRE), 12-5(VSE), 09-9 (VRE)) and compared with the controls, echinomycin and levofloxacin. Compound **1** exhibited moderate antibacterial activities with MIC values of 16–64 μg/mL against not only the antibiotic-sensitive but also the antibiotic-resistant bacteria. Its activities were lower than those of echinomycin (Table 5).

Meanwhile, compound **1** was also evaluated for anti-tumor activity *in vitro* against 12 human tumor cell lines (HCT-116, HepG2, BGC-823, NCI-H1650, A2780, SW1990, Mia-PaCa-2, U87 MG, SK-N-SH, Jurkat, ACHN, 786-O) by MTT method and echinomycin was used as the control. Compound **1** displayed strong anti-tumor activity against the tested cell lines ACHN, 786-O, U87 MG and Jurkat with the IC_50_ values of 0.552, 0.721, 0.627, and 0.414 μM, respectively. Unfortunately, its activities were also lower than those of echinomycin (Table 6).

**Table 5:** Minimum inhibitory concentrations (MIC) for compound **1** and echinomycin.

	MIC (μg/mL)														
Microorganism	*Staphylococcus epidermidis*			*Staphylococcus aureus*					*Enterococcu faecium*			*Enterococcus faecalis*			
Strain No.	ATCC 12228	12-6	12-8	ATCC 29213	ATCC 33591	15	12-28	12-33	ATCC 700221	12-1	12-3	ATCC 29212	ATCC 51299	12-5	09-9
Phenotype	MSSE ^a^	MSSE	MRSE ^b^	MSSA ^c^	MRSA ^d^	MSSA	MSSA	MRSA	VRE ^e^	VRE	VSE ^f^	VSE	VRE	VSE	VRE
Compound **1**	32	32	32	32	32	32	32	32	32	32	64	16	16	16	16
Echinomycin	0.5	0.25	0.5	0.5	0.5	0.5	0.25	0.5	0.5	0.5	0.5	0.25	0.5	0.25	0.25
Levofloxacin (conrol)	0.25	0.5	4	0.125	0.25	0.125	0.25	64	128	128	64	0.5	1	1	>128

Notes: ^a^ Methicillin-susceptible *Staphylococcus epidermidis*; ^b^ Methicillin-resistant *Staphylococcus epidermidis*; ^c^ Methicillin-susceptible *Staphylococcus aureus*; ^d^ Methicillin-resistant *Staphylococcus aureus*; ^e^ Vancomycin-susceptible *Enterococcus*; ^f^ Vancomycin-resistant *Enterococcus*.

**Table 6:** Anti-tumor activities (IC_50_, μM) of compound **1** and echinomycin.

Compounds	IC_50_ (μM)											
	ACHN	SW1990	Mia-PaCa-2	786-O	U87 MG	SK-N-SH	Jurkat	HCT-116	NCI-H1650	HepG2	BGC-823	A2780
Compound **1**	0.552	2.560	4.750	0.721	0.627	5.174	0.414	8.61	3.90	>10	>10	>10
Echinomycin	<0.0032	0.0026	<0.0032	<0.0032	<0.0032	0.027	<0.0032	<0.01	<0.01	<0.01	<0.01	<0.01

## 3. Experimental Section

### 3.1. General

Optical rotations were measured on a JASCO P-2000 digital polarimeter (JASCO Corporation Tokyo, Japan). UV measurements were recorded using an Agilent Cary 300 spectrometer (Agilent Technologies, Santa Clara, United States). ^1^H and ^13^C NMR, and 2D NMR spectra were obtained at 500 and 125 MHz, using a Bruker AVANCE 500-III spectrometer (Bruker Biospin Group, Karlsruhe, Germany) in chloroform or DMSO-*d*_6_ with TMS as an internal reference. HR-ESI-MS data were measured using an Agilent 1100 LC/MSD Trap SL LC/MS/MS spectrometer (Agilent Technologies, Santa Clara, CA, USA). Semipreparative HPLC was performed by an HPLC system equipped with a Shimadzu LC-6AD pump and a Shimadzu SPD-20A prominence UV-VIS detector (Shimadzu Corporation, Kyoto, Japan) using an Agilent C_18_ column (5 μM, 250 × 9.6 mm). Column chromatography was performed with silica gel (200-300 mesh, Qingdao Marine Chemical Ltd., Qingdao, China), RP-18 (40–60 μM, GE healthcare, Fairfield, CT, USA), and Sephadex LH-20 (18–110 μM, GE healthcare, Fairfield, CT, USA)

### 3.2. Bacterial Material and Fermentation

The producing strain LS298 was isolated from a sponge *Gelliodes carnosa* collected from Lingshui Bay, Hainan Province of China near Xincun Harbor (18°24′5.49″ N, 109°59′37.76″ E), in August 2007 [9]. It was identified as *Streptomyces* sp. on the basis of the morphology and 16S rRNA gene sequence analysis by comparison with other sequences in the GenBank database. The DNA sequence was deposited in GenBank (Accession No. FJ937945). The strain LS298 was first cultivated on Gause I agar plates (Gause I: starch 20 g; KNO_3_ 1 g; NaCl 0.5 g; K_2_HPO_3_ 0.5 g; MgSO_4_ 0.01 g; Natural seawater 1 L; pH 7.0–7.2) at 28 °C for three days. Then, the mycelia were inoculated into 500 mL Erlenmeyer flasks, each containing 100 mL of liquid A1 medium (A1: starch 10 g; Yeast extract 4 g; Peptone 2 g; Natural seawater 1 L; pH 7.0–8.0). The flasks were incubated at 28 °C on a rotary shaker (200 rpm) for three days. Seed culture (10 mL) was transferred into three hundred 500 mL Erlenmeyer flasks (each Erlenmeyer flask contained 100 mL A1 medium) and incubated at 28 °C on a rotary shaker (200 rpm) for nine days.

### 3.3. Extraction and Isolation

The culture broth (30 L) was repeatedly extracted with ethyl acetate (*v*/*v* 1:3, three time) by ultrasound, and the organic solvent was evaporated to dryness under a vacuum to afford the crude extract (3.5 g). The crude extract was first subjected to reversed-phase (RP) column chromatography (4 × 60 cm, 200 g) using a decreasing polar aqueous CH_3_OH/H_2_O (20%, 50%, 80%, and 100%, *v*/*v*, each 2 L) gradient elution and afforded four primary fractions (A1–A4). Fraction A4 (100% CH_3_OH, 920 mg) was separated again by Sephadex LH-20 chromatography (2 × 100 cm, 80 g) using 1:10 CHCl_3_/CH_3_OH (each 20 mL) as eluent. Then, the fractions were combined to four fractions (B1–B4) on the basis of their HPLC behavior. Purification of the B2 subfractions (100 mg) by semipreparative HPLC afforded compound **1** (10.0 mg, 55% CH_3_CN in H_2_O, flow rate 4 mL/min, t_R_ = 37.8 min). Fraction A1 (20% CH_3_OH/H_2_O, 715 mg) was separated via Sephadex LH-20 (2 × 100 cm, 80 g) using CH_3_OH (each 20 mL) as eluent. The fractions were examined by HPLC and the Fraction A1.5 to A1.8 were combined, then it was purified by semipreparative HPLC to provide compound **2** (8.2 mg, 36% CH_3_CN in H_2_O, flow rate 4 mL/min, t_R_ = 27.3 min). Fraction A3 (80% CH_3_OH/H_2_O, 530 mg) was further separated on RP chromatography (3 × 40 cm, 100 g) eluted with CH_3_OH/H_2_O (60%, 70%, 80%, 100%, *v*/*v*, each 1 L) and fraction A3.3 (80% CH_3_OH, 50 mg) was purified by Sephadex LH-20 chromatography (2 × 100 cm, 80 g) eluted with CH_3_OH to get the yellow band compound **3** (7.5 mg). Fraction A2 (50% CH_3_OH/H_2_O, 450 mg) was further separated by semipreparative HPLC to provide compounds **4** (8.0 mg, 42% CH_3_CN in H_2_O, flow rate 4 mL/min, t_R_ = 31.5 min)) and **5** (7.8 mg, 47% CH_3_CN in H_2_O, flow rate 4 mL/min, t_R_ = 42.6 min).

Compound **1**: amorphous yellow powder; ${\left[\alpha \right]}_{D}^{\mathrm{25}}$ −3.8 (*c* 0.20, CHCl_3_); UV (CHCl_3_) λ_max_ (log ε) 245.2 (2.6), 325.8 (1.9); ^1^H and ^13^C NMR data, see Table 1; HRESIMS *m*/*z* 1101.4288 [M + H]^+^ (calcd for C_51_H_65_N_12_O_12_S_2_, 1101.4286).

Compound **2**: white powder; ${\left[\alpha \right]}_{D}^{\mathrm{25}}$ −15.0 (*c* 0.20, CH_3_OH); UV (CH_3_OH) λ_max_ (log ε) 240.2 (3.6); ^1^H and ^13^C NMR data, see Table 3; HRESIMS *m*/*z* 249.1209 [M + Na]^+^ (calcd for C_11_H_18_N_2_O_3_Na, 249.1210).

### 3.4. Hydrolysis of Compounds **1**–**2** and HPLC Analysis by Marfey’s Method

Compounds **1** (1.0 mg) and **2** (1.4 mg) were dissolved in 6 N HCl (1 mL), and heated at 110 °C for 18 h. After cooling to room temperature, the hydrolysates were dried under reduced pressure and resuspended into 100 μL of H_2_O.Then they were treated with 1 M NaHCO_3_ (25 μL), and reacted with 100 μL of 1% (*w*/*v*) FDAA in acetone at 40 °C for 1.5 h. After cooling to room temperature, the mixture was added with 1 M HCl (25 μL) to neutralize and terminate the reaction. MeOH was then added to the quenched reaction to afford a total volume of 500 μL; 10 μL of each hydrolysate derivatization reaction was used for HPLC analysis using an Agilent C_18_ column (150 × 4.6 mm, 5 μM) with a solvent gradient from 15% to 45% solvent B (solvent A: CH_3_COOH/H_2_O, 0.05/99.95, solvent B: CH_3_CN) over the course of 30 min and UV detection at 340 nm at a flow rate of 1 mL/min. Similarly, 10 μL of the standard amino acids in H_2_O (4 μM) were added to 1 M NaHCO_3_ (20 μL) and each mixture was treated with 1% (*w*/*v*) FDAA (50 μL) for 1.5 h at 40 °C. Derivatization reactions were terminated with 1 M HCl (20 μL) and diluted to a total volume of 500 μL with MeOH. Of these standard amino acid derivatization reactions, 10 μL was subjected to HPLC analysis and used as structural standards in the elucidation of structures **1** and **2**.

### 3.5. Biological Assays

Antibacterial and anti-tumor assays were performed with compounds of purity >90% by HPLC.

#### 3.5.1. Antibacterial Activity

The testing bacteria used in this study were as follows: *Staphylococcus epidermidis* (ATCC 12228 (MSSE), 12-6 (MSSE), 12-8 (MRSE)), *Staphylococcus aureus* (ATCC 29213 (MSSA), ATCC 33591 (MRSA), 15 (MSSA), 12-28 (MSSA), 12-33 (MRSA)), *Enterococcus faecium* (ATCC 700221 (VRE), 12-1 (VRE), 12-3 (VSE)), *Enterococcus faecalis* (ATCC 292121 (VSE), ATCC 51299 (VRE), 12-5 (VSE), 09-9 (VRE)), which included strains from the ATCC collection and clinical isolates. MIC values against the 15 bacterial strains for compound **1** were measured by using the agar dilution method described by the Clinical Laboratory Standards Institute [26]. Briefly, the test medium was Mueller-Hinton broth, and the inoculum was 10,000 colony forming units (CFU)/spot. The compound 1 was incorporated into the agar medium, with each plate containing a different concentration of the compound. Culture plates were incubated at 35 °C for 18 h, and MICs were then recorded. The positive controls were levofloxacin and echinomycin. The final concentrations of compounds ranged from 0.03 to 128 μg/mL. The MIC was defined as the lowest concentration that prevented visible growth of the bacteria [27].

#### 3.5.2. Anti-Tumor Assay

The anti-tumor activities of the new compound **1** and echinomycin against 12 tumor cell lines including: human colonic carcinoma (HCT-116), human hepatoma (HepG2), human gastric cancer (BGC-823), human non-small cell lung cancer (NCI-H1650), human ovarian cancer (A2780), human pancreatic cancer (SW1990, Mia-PaCa-2), human multiform glioblastoma (U87 MG), Human neuroblastoma (SK-N-SH), human T-cell leukemia (Jurkat) and human renal clear cell carcinoma (ACHN, 786-O) were determined by MTT method [28,29,30,31].

The human colonic carcinoma (HCT-116), human hepatoma (HepG2), human gastric cancer (BGC-823), human non-small cell lung cancer (NCI-H1650), human ovarian cancer (A2780), human pancreatic cancer (SW1990, Mia-PaCa-2), human multiform glioblastoma (U87 MG), human neuroblastoma (SK-N-SH), human renal clear cell carcinoma (ACHN, 786-O) were maintained in DMEM medium; human T-cell leukemia (Jurkat) was maintained in RPMI 1640 medium. Both media were supplemented with 10% heat inactivated fetal bovine serum, 100 units/mL of penicillin and 100 μg/mL of streptomycin, in a humidified 5% CO_2_/air atmosphere at 37 °C. MTT assay: briefly, logarithmic cells were digested with 0.25% pancreatic enzyme-EDTA and plated in the 96-well plates at concentration of 800–2000/100 μL per well. Compounds at final concentrations of 0.5 to 50 μg/mL were added with triplicates of each concentration after 24 h. The cells were incubated further at 37 °C for 96 h, the medium was aspirated, and 100 μL MTT of 0.5 mg/mL in medium was added. After 4h incubation, the medium was aspirated and 200 μL DMSO was added to solubilize the formazan crystals. Absorbance of the converted dye was measured at a wavelength of 570 nm with background subtraction at 650 nm. The dose-response curves were fitted with Sigma plot and IC_50_s were determined.

## 4. Conclusions

In summary, a novel echinomycin analogue quinomycin G (**1**), a new cyclic dipeptide cyclo-(l-Pro-4-OH-l-Leu) (**2**), along with three known antibiotics tirandamycin A (**3**), tirandamycin B (**4**) and staurosporine (**5**), were isolated and characterized from the marine *Streptomyces* sp. LS298. To our knowledge, this was the first time to obtain three types of antibiotics from one strain from the same batch, though these types of antibiotics have been isolated from different strains of genus *Streptomyces* [18,32]. What is more, the 1-keto-4′-enol form of tirandamycin B was reported firstly, and the trend in NMR data of keto-enol tautomers in tirandamycins was discussed.

Compound **1** exhibited moderate antibacterial and remarkable anti-tumor activities; however, its activities were lower than those of echinomycin, which indicated that the bicyclic peptide of these compounds was required for their activities. Echinomycin has been studied for many years and the mechanism of its antibacterial and antitumor activities is considered to be DNA bis-intercalation. Due to the similar structure, we proposed that compound **1** has a similar mechanism against the bacteria and the tumor cells. Efforts are underway to discover novel and potential echinomycin analogues in future work through combinations of genome mining and heterologous expression approaches.

## Supplementary Files

Supplementary File 1
