# Supplementary material for: A New Analogue of Echinomycin and a New Cyclic Dipeptide from a Marine-Derived Streptomyces sp. LS298
Source: Mar Drugs. 2015 Nov 18;13(11):6947–61. doi: 10.3390/md13116947 (PMC4663560; doi:10.3390/md13116947)
Supplement: Supplementary File 1 [file marinedrugs-13-06947-s001.docx]

Supplementary Materials


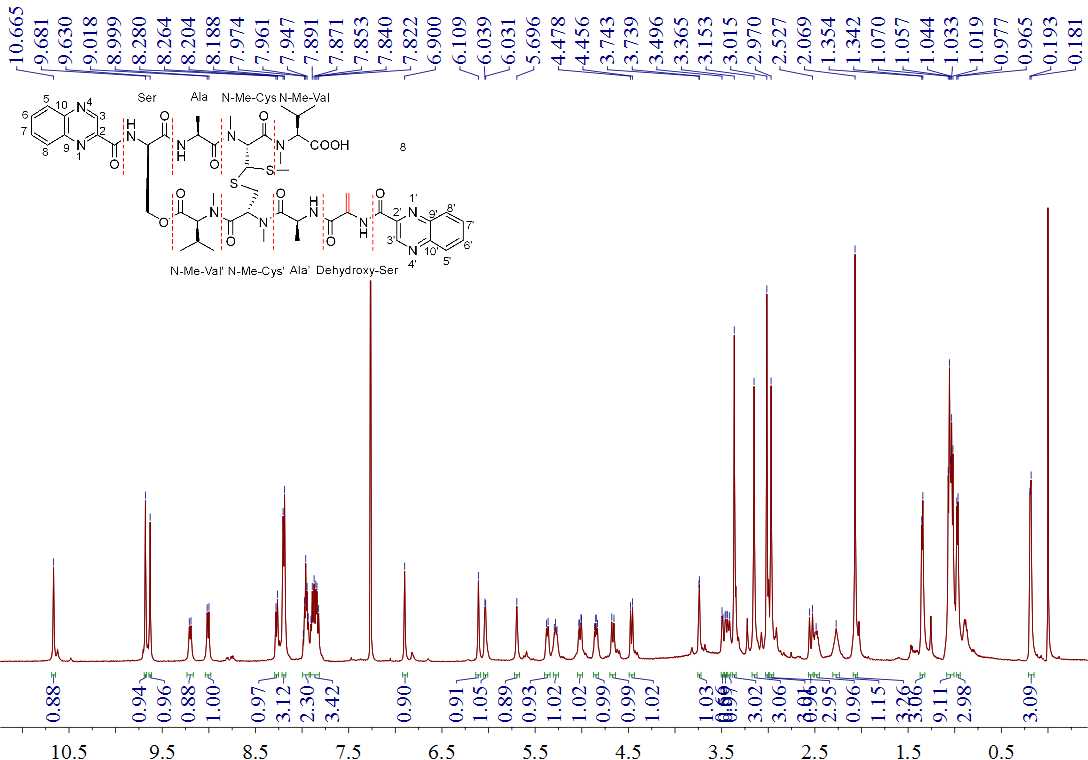


**Figure S1.** The ^1^H NMR spectrum of quinomycin G (**1**) in CDCl_3_.


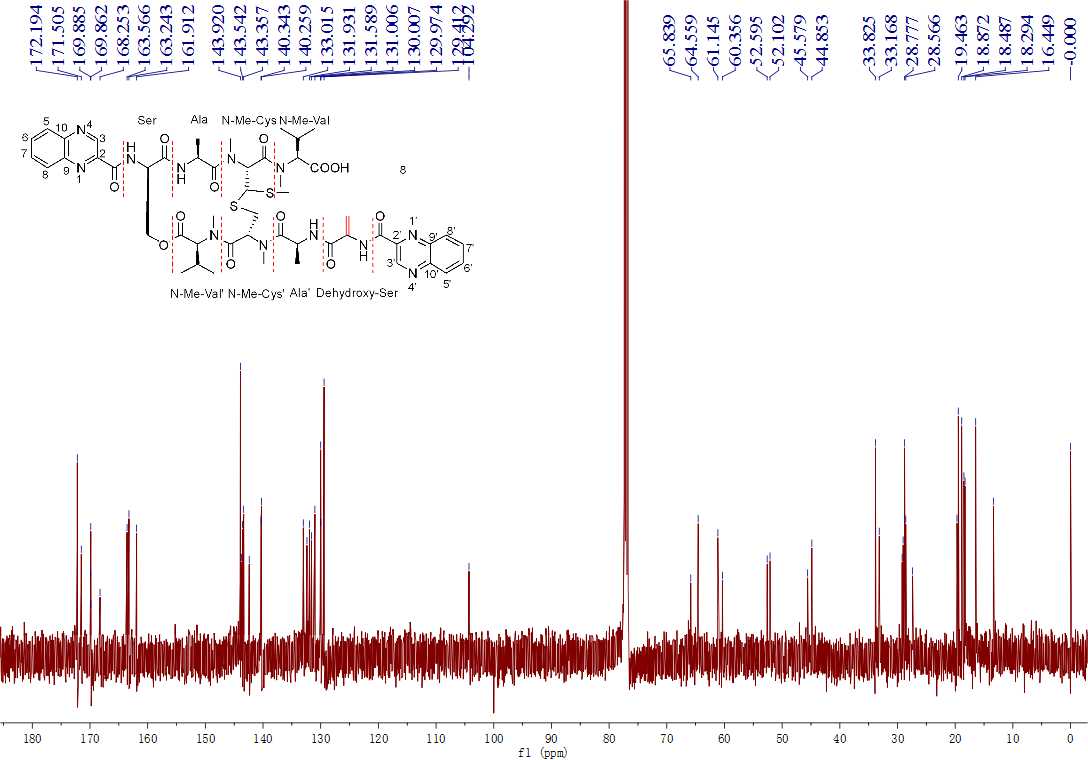


**Figure S2.** The ^13^C NMR spectrum of quinomycin G (**1**) in CDCl_3_.


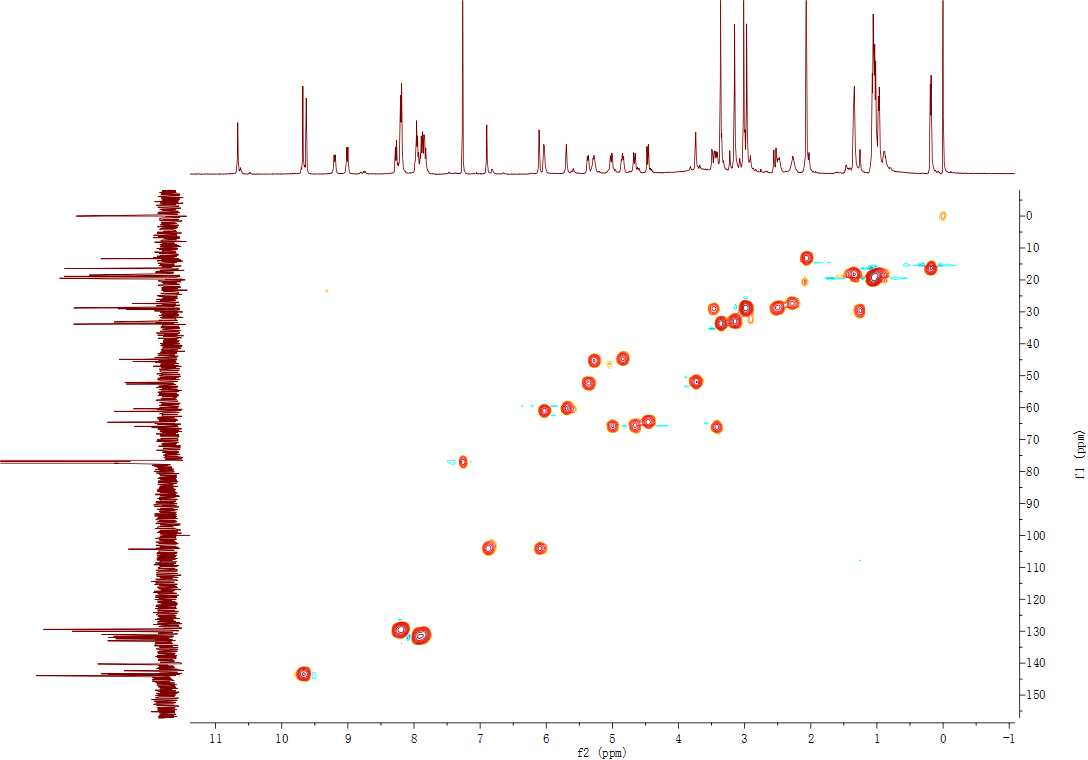


**Figure S3.** The HSQC spectrum of quinomycin G (**1**) in CDCl_3_.


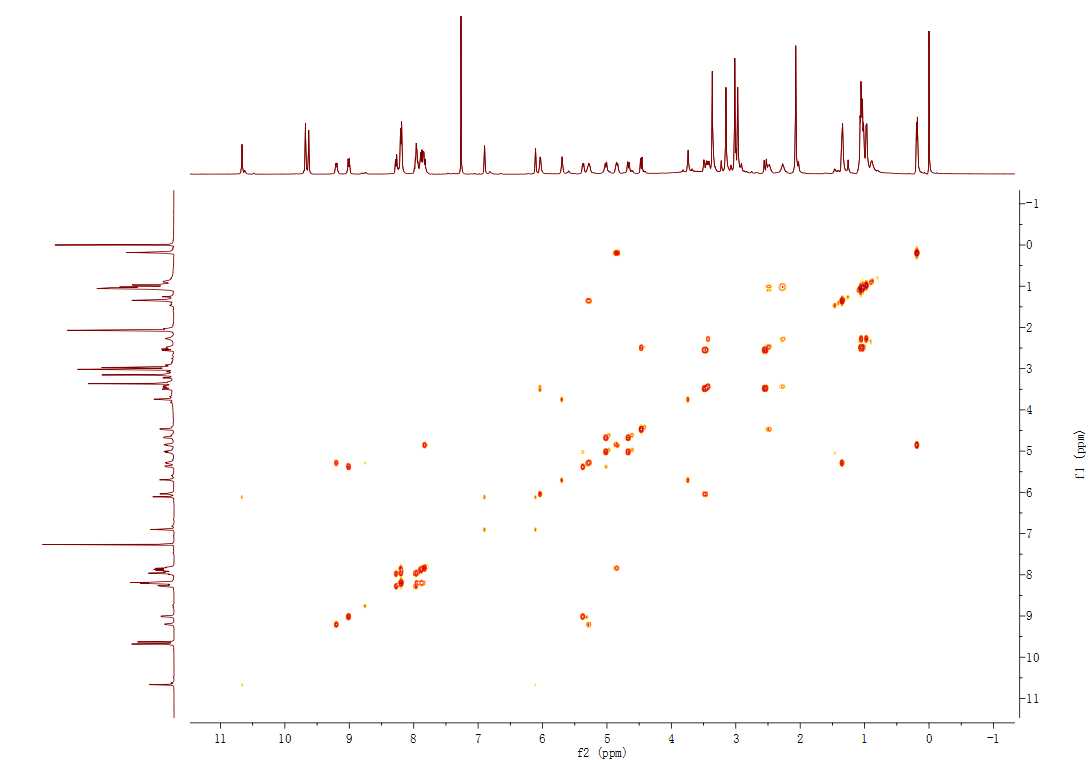


**Figure S4.** The ^1^H-^1^H COSY spectrum of quinomycin G (**1**) in CDCl_3_.


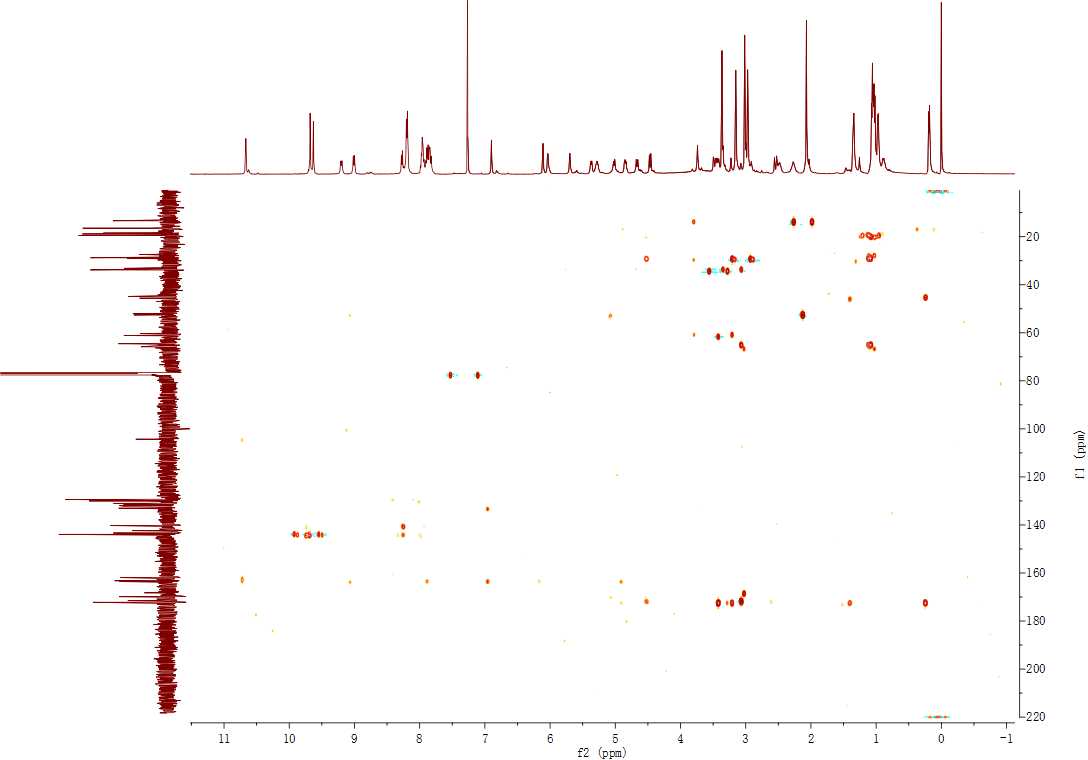


**Figure S5.** The HMBC spectrum of quinomycin G (**1**) in CDCl_3_.


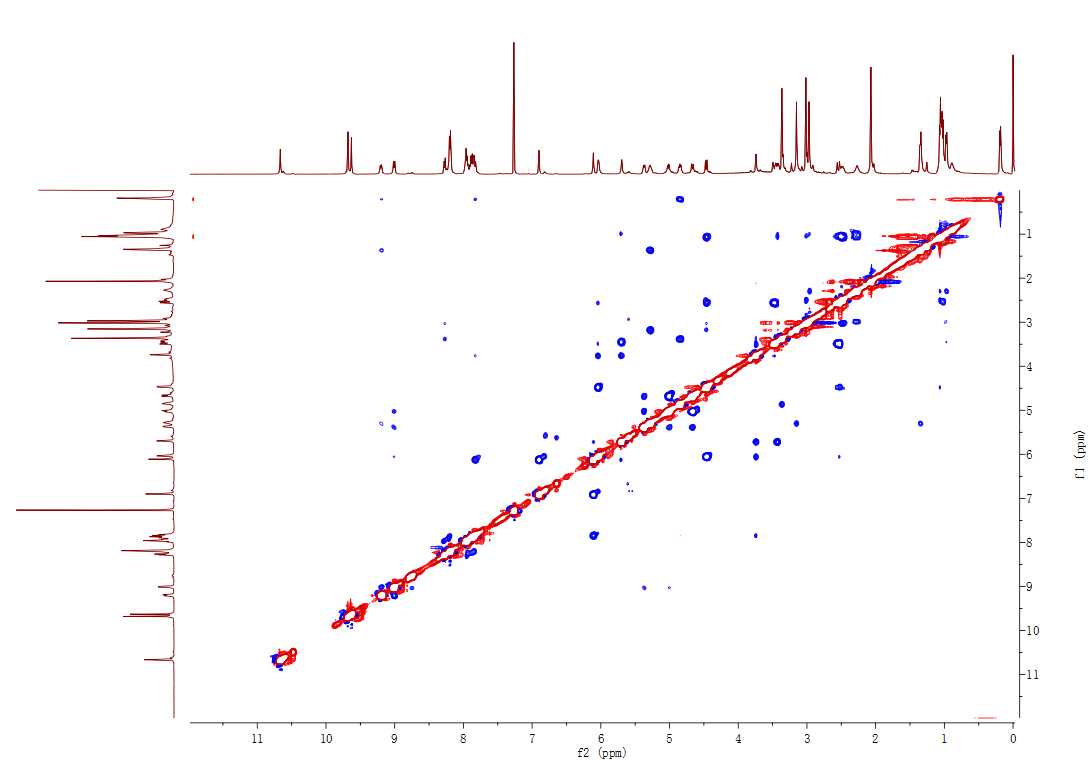


**Figure S6.** The ROESY spectrum of quinomycin G (**1**) in CDCl_3_.


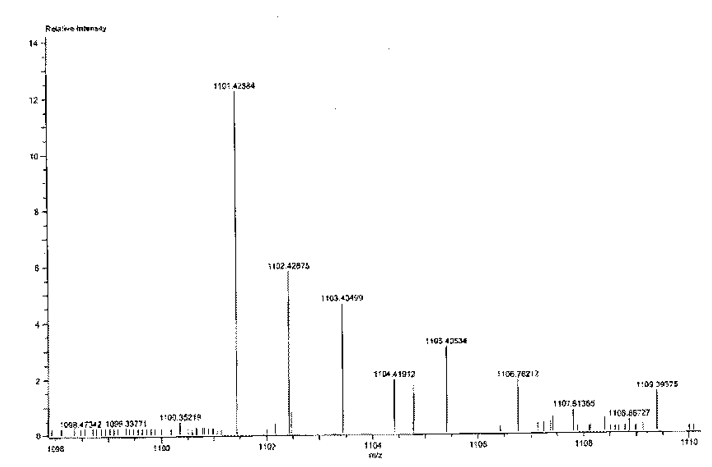


__

**Figure S7.** The HRESIMS spectrum of quinomycin G (**1**).

**Figure S8.** The key ^1^H-^1^H COSYcorrelations of quinomycin G (**1**).


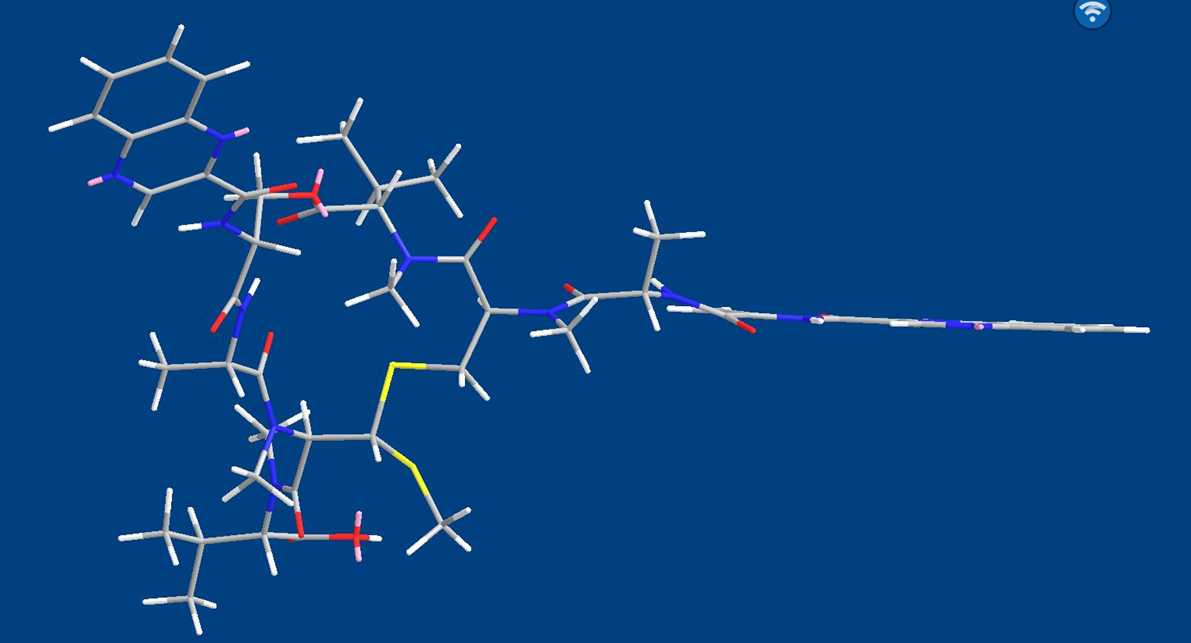


**Figure S9.** The Chem3D MM2 energy minimization model of quinomycin G (**1**).


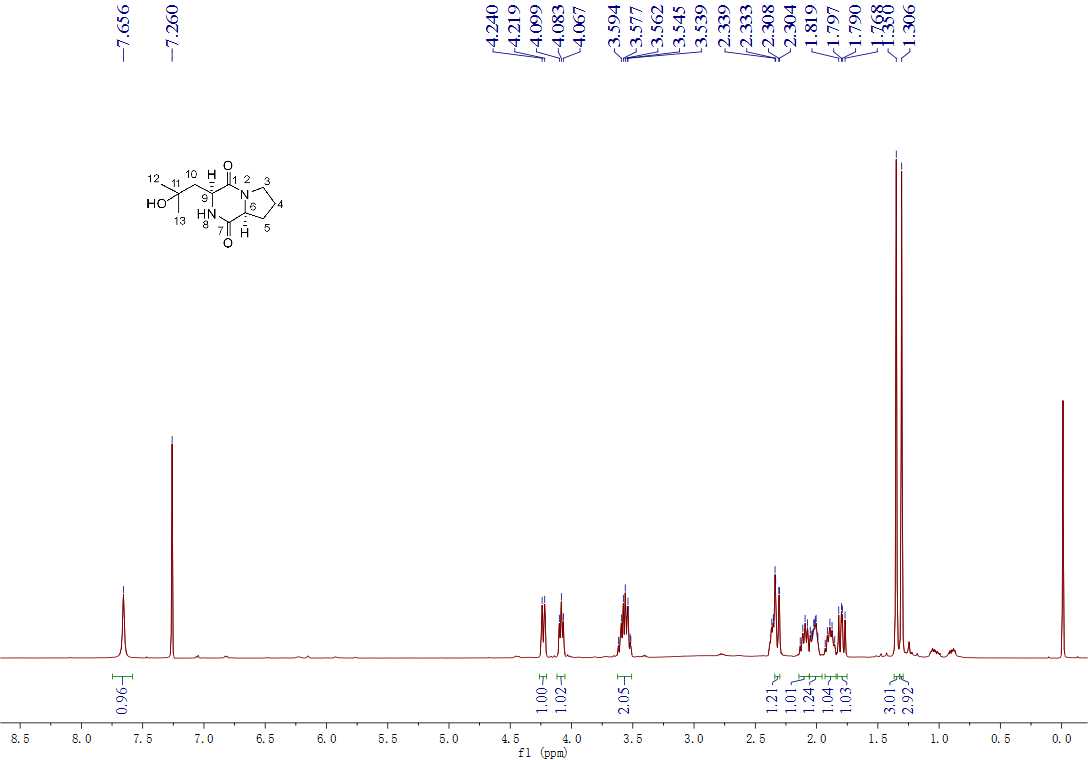


**Figure S10.** The ^1^H NMR spectrum of cyclo-(l-Pro-4-OH-l-Leu) (**2**) in CDCl_3_.


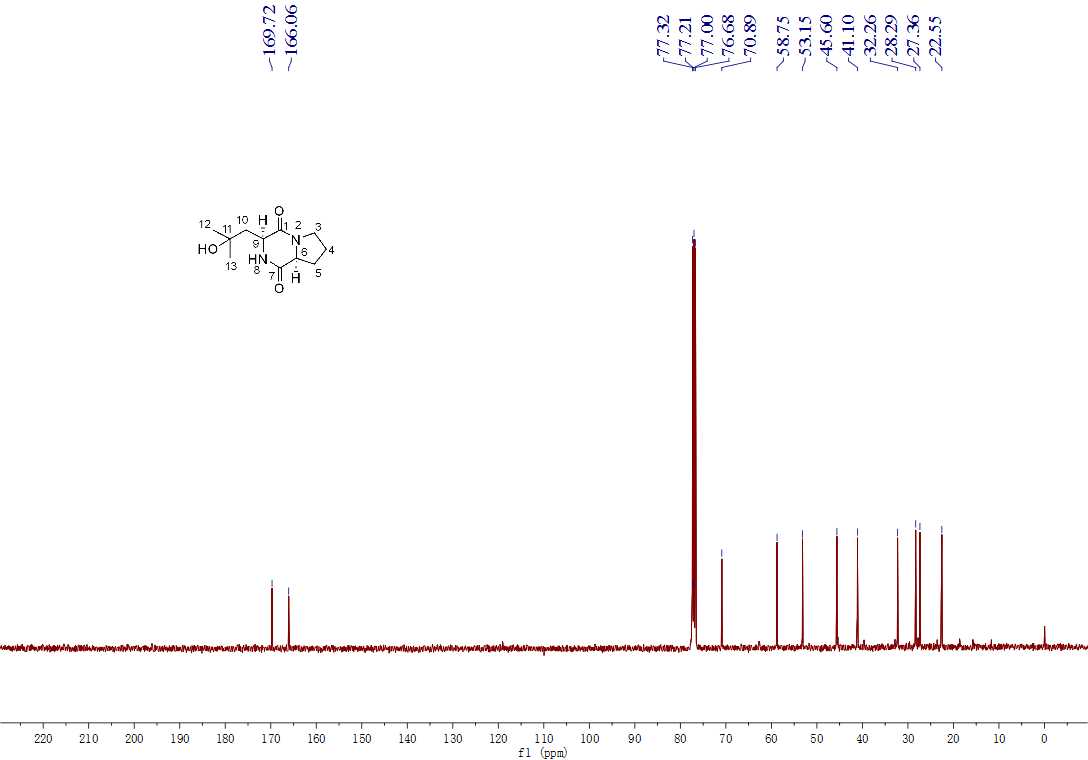


**Figure S11.** The ^13^C NMR spectrum of cyclo-(l-Pro-4-OH-l-Leu) (**2**) in CDCl_3_.

_
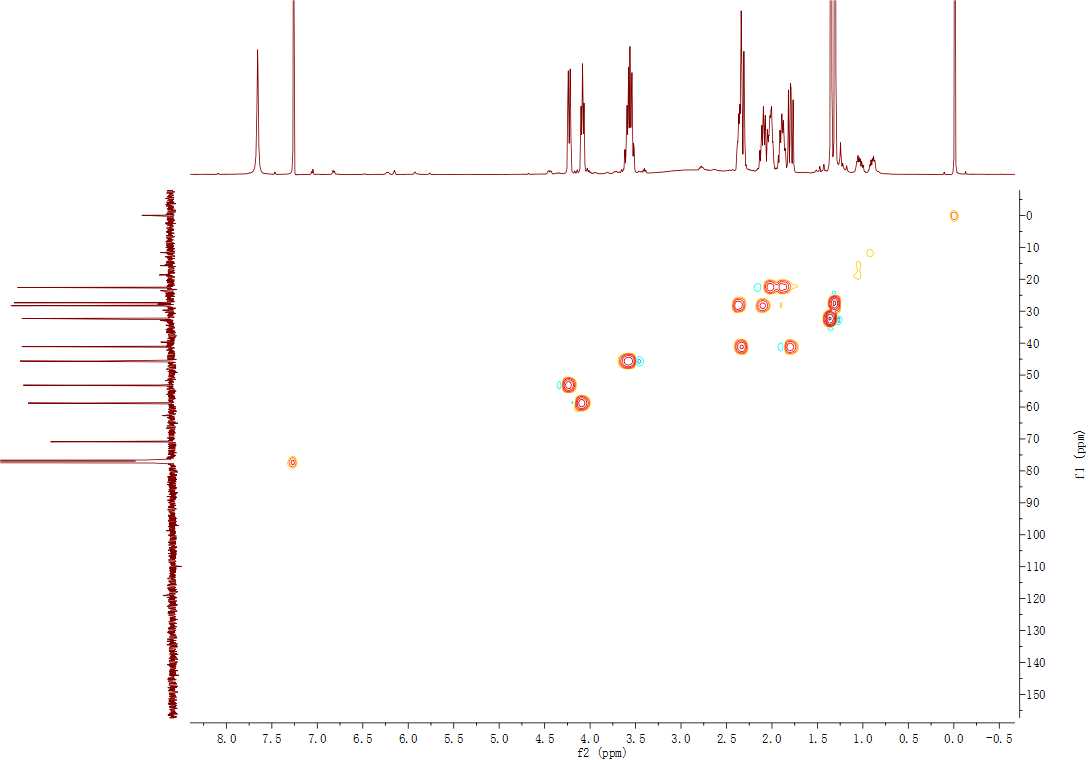
_

**Figure S12.** The HSQC spectrum of cyclo-(l-Pro-4-OH-l-Leu) (**2**) in CDCl_3_.


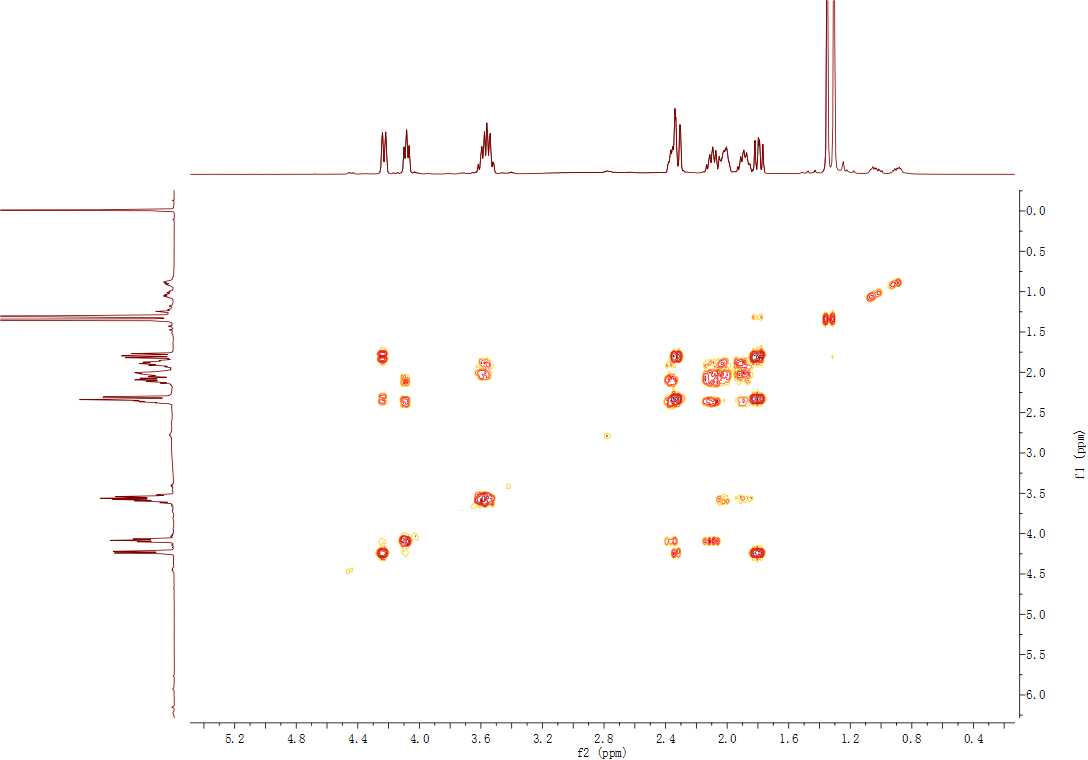


**Figure S13.** The ^1^H-^1^H COSY spectrum of cyclo-(l-Pro-4-OH-l-Leu) (**2**) in CDCl_3_.


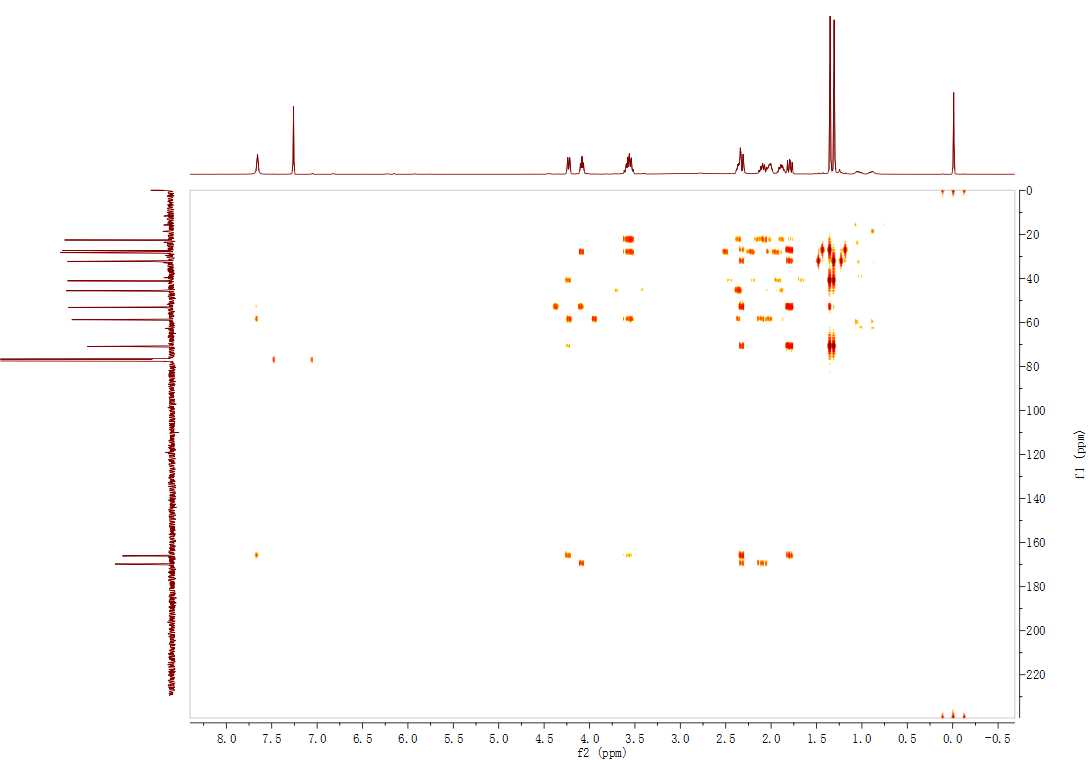


**Figure S14.** The HMBC spectrum of cyclo-(l-Pro-4-OH-l-Leu) (**2**) in CDCl_3_.

_
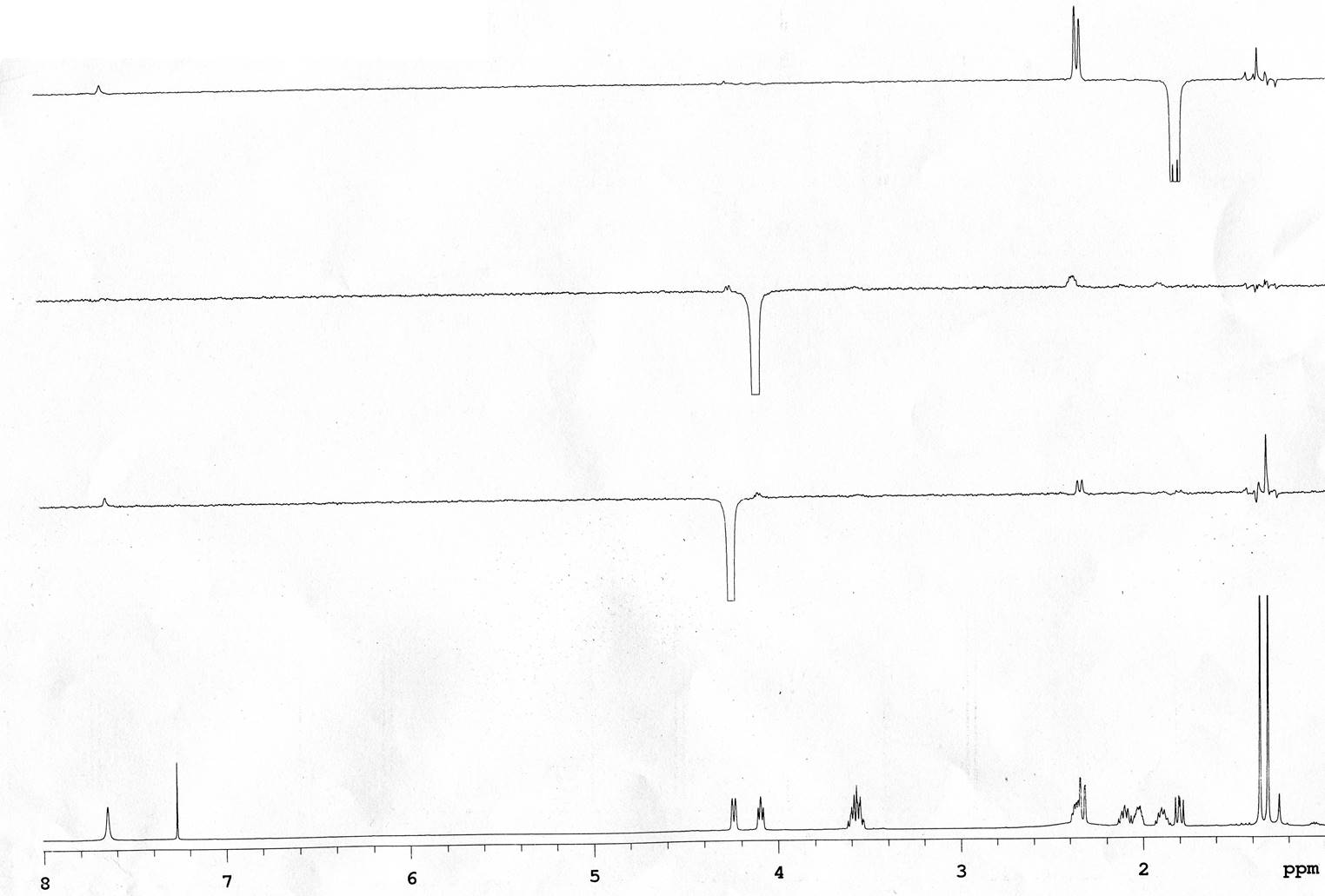
_

**Figure S15.** The NOE spectrum of cyclo-(l-Pro-4-OH-l-Leu) (**2**) in CDCl_3_.


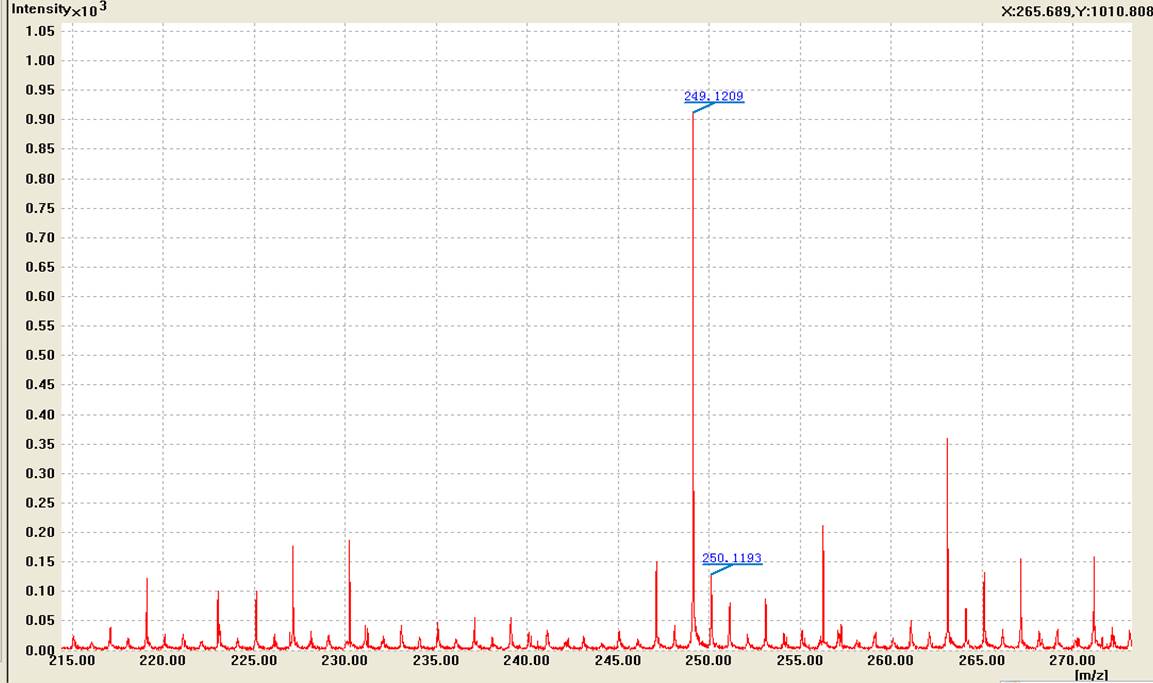


| **Mass** | **Calc. Mass** | **Mass Difference (ppm)** | **^12^C** | **^1^H** | **^14^N** | **^16^O** | **^23^Na** |
| --- | --- | --- | --- | --- | --- | --- | --- |
| 249.1209 | 249.1210 | 0.40 | 11 | 18 | 2 | 3 | 1 |

**Figure S16.** The HRESIMS spectrum of cyclo-(l-Pro-4-OH-l-Leu) (**2**).


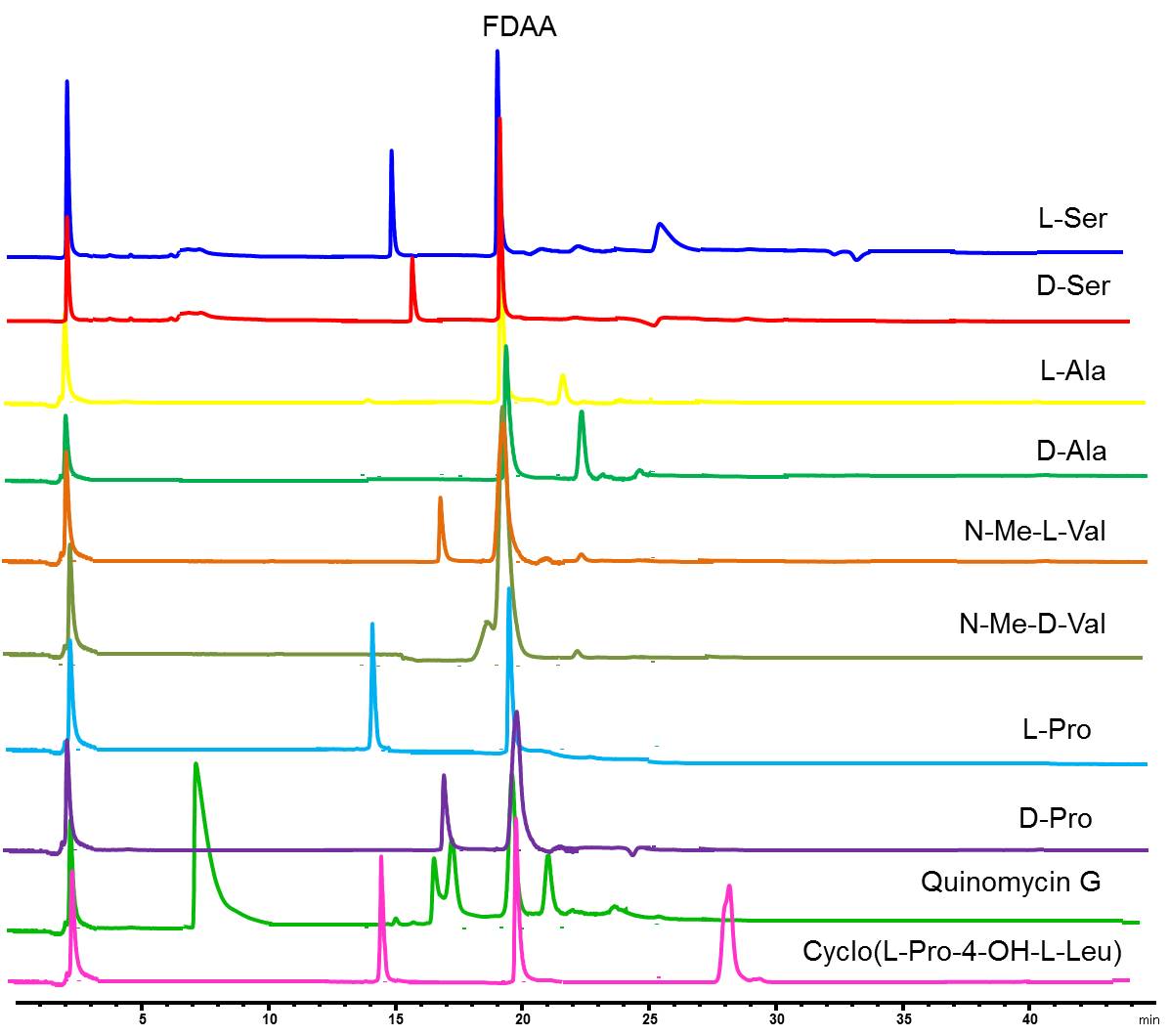


**Figure S17.** The chromatograms of FDAA derivatives of the hydrolysates of compounds **1**–**2** and amino acids standards.


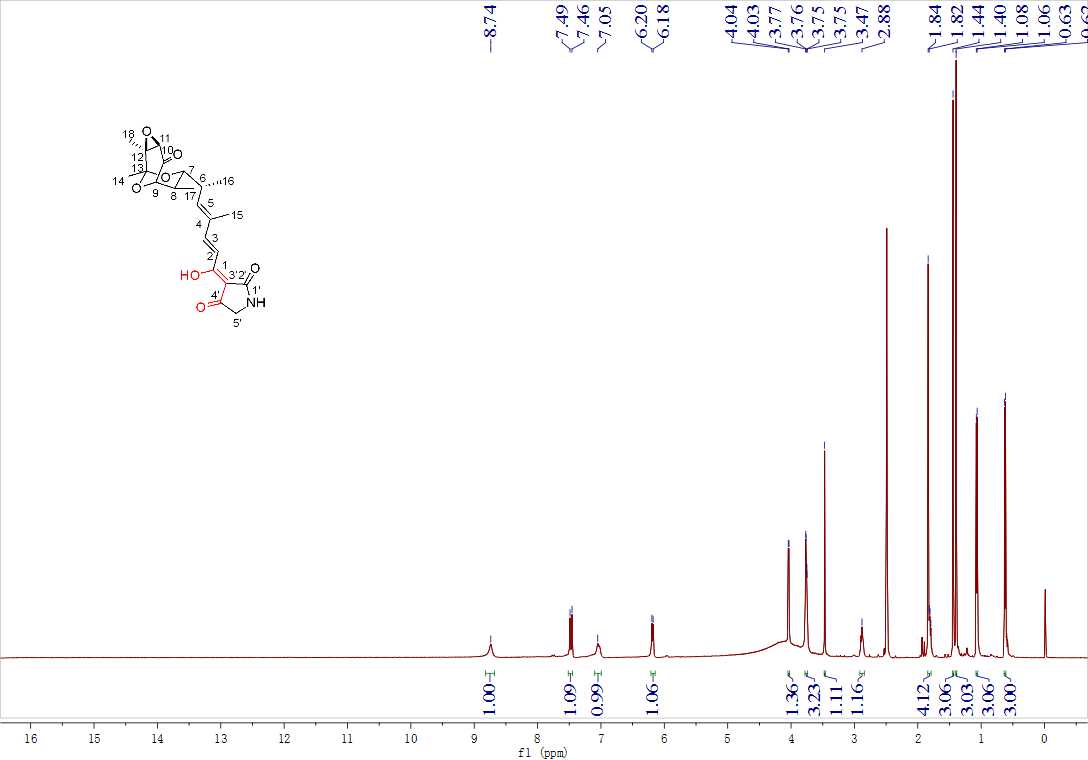


**Figure S18.** The ^1^H NMR spectrum of tirandamycin A (**3**) in DMSO-*d*_6_.


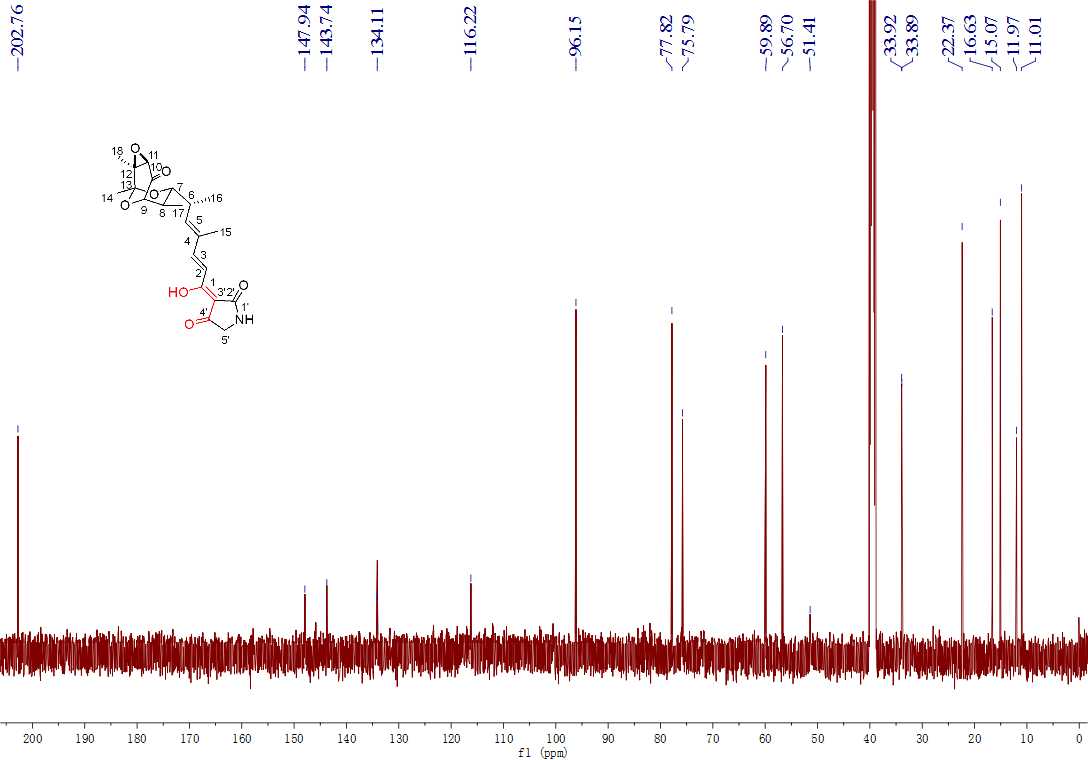


**Figure S19.** The ^13^C NMR spectrum of tirandamycin A (**3**) in DMSO-*d*_6_.

_
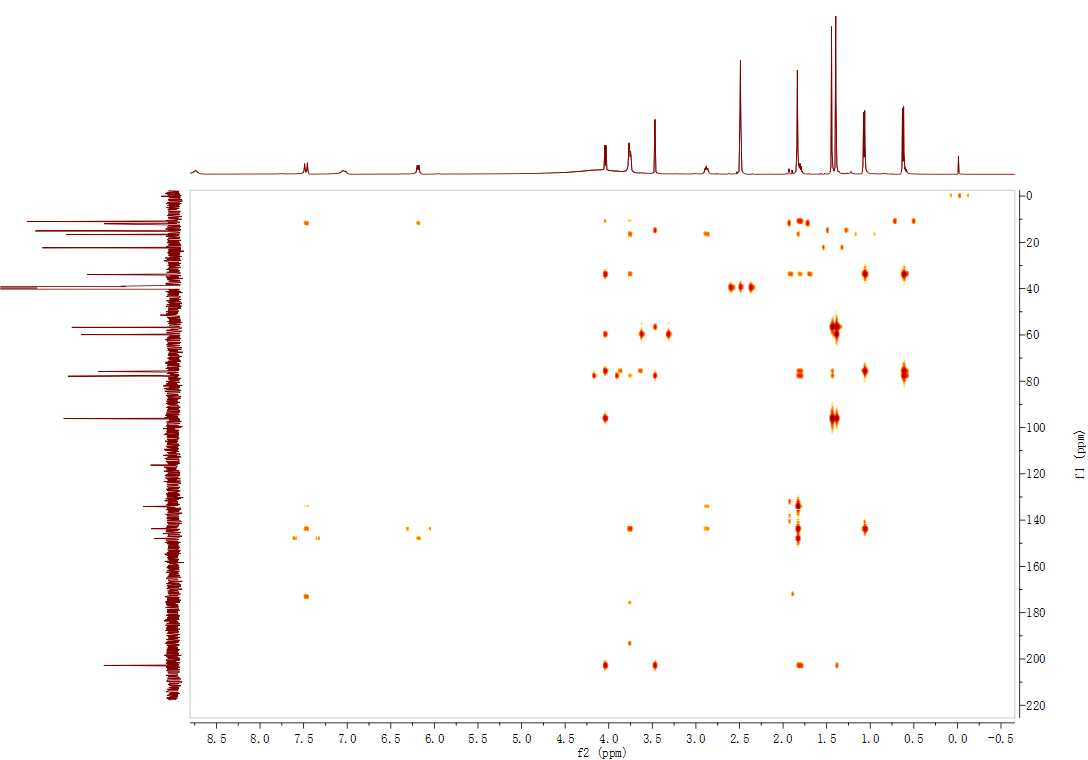
_

**Figure S20.** The HMBC spectrum of tirandamycin A (**3**) in DMSO-*d*_6_.


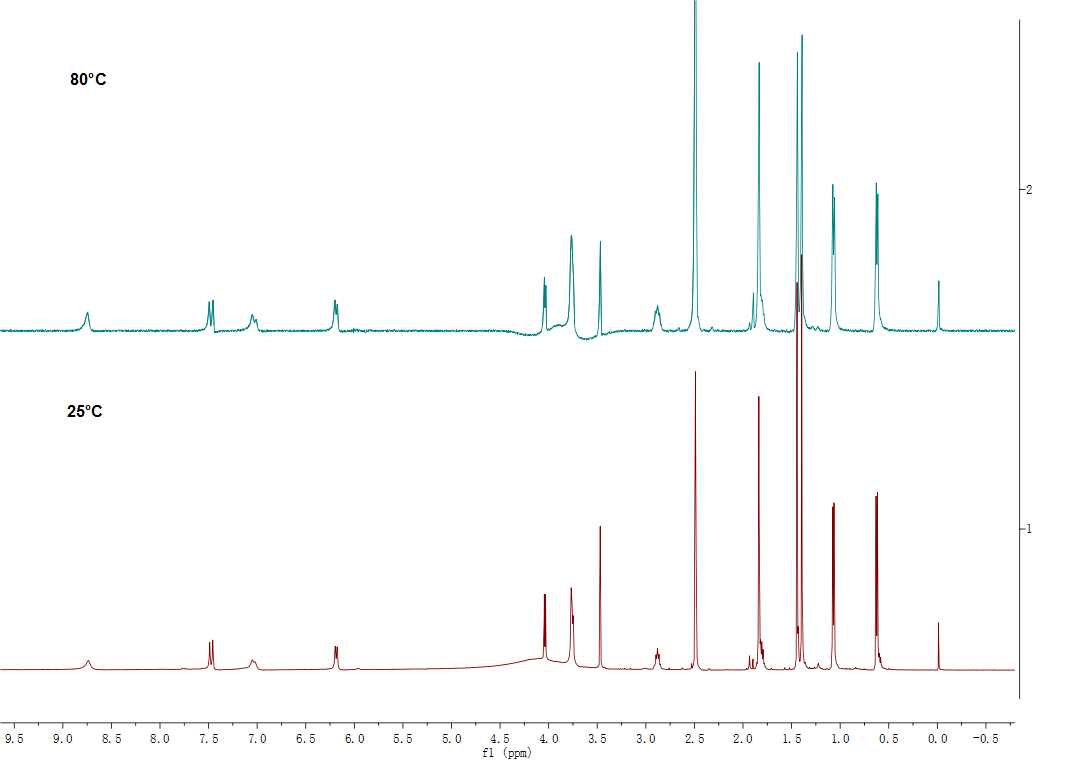


**Figure S21.** The variable temperature experiment for ^1^H-NMR of tirandamycin A (**3**).


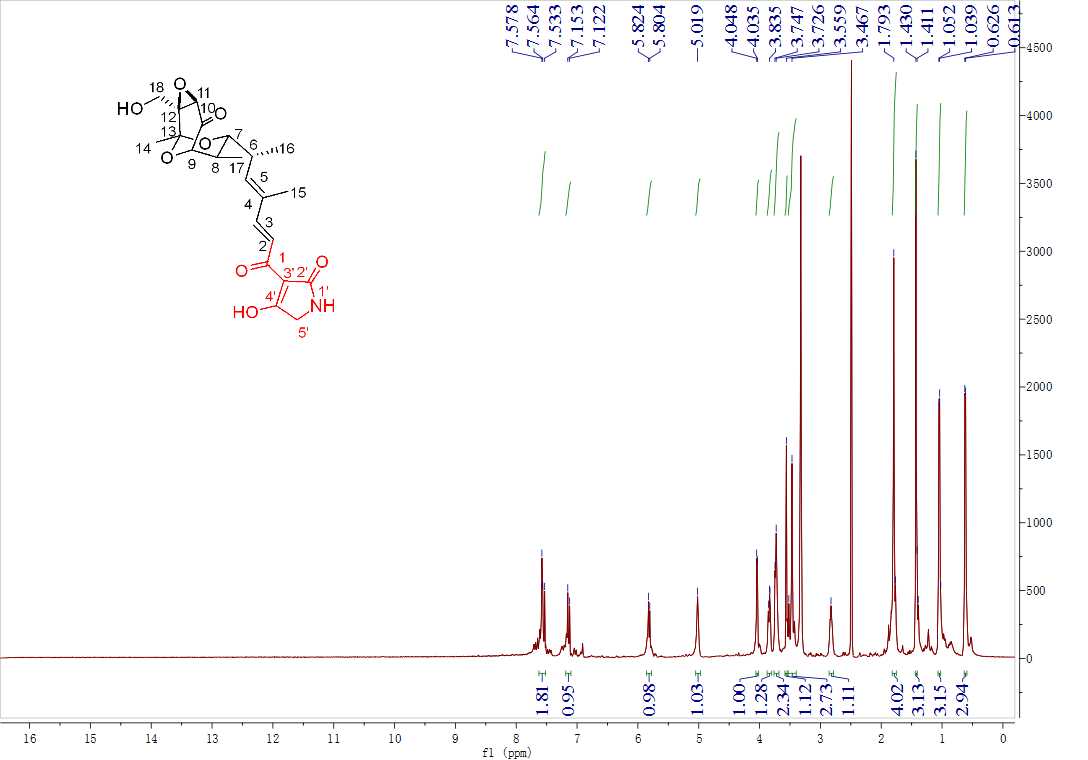


**Figure S22.** The ^1^H NMR spectrum of tirandamycin B (**4**) in DMSO-*d*_6_.


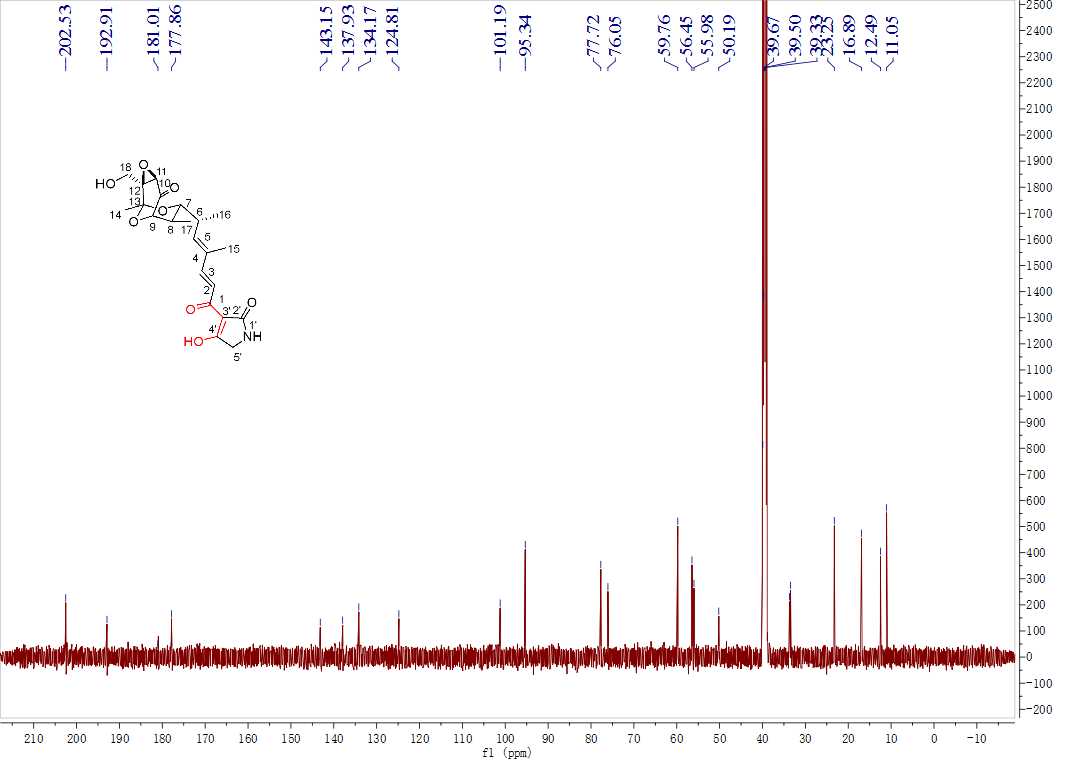


**Figure S23.** The ^13^C NMR spectrum of tirandamycin B (**4**) in DMSO-*d*_6_.


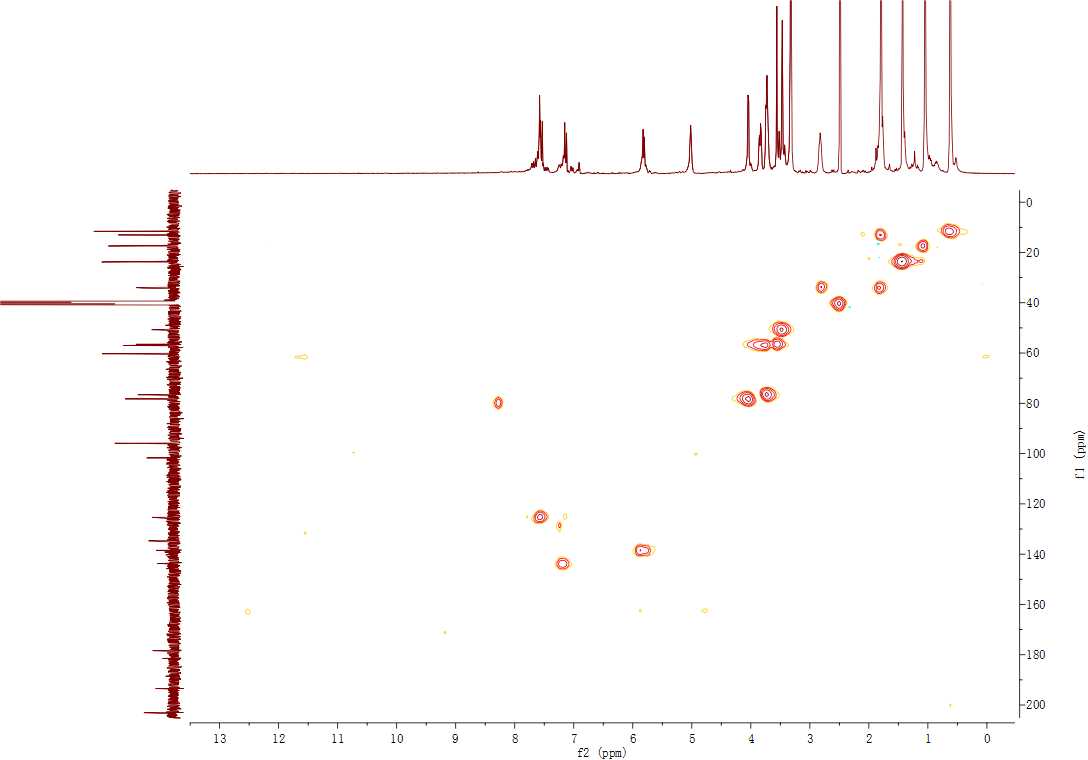


**Figure S24.** The HSQC spectrum of tirandamycin B (**4**) in DMSO-*d*_6_.


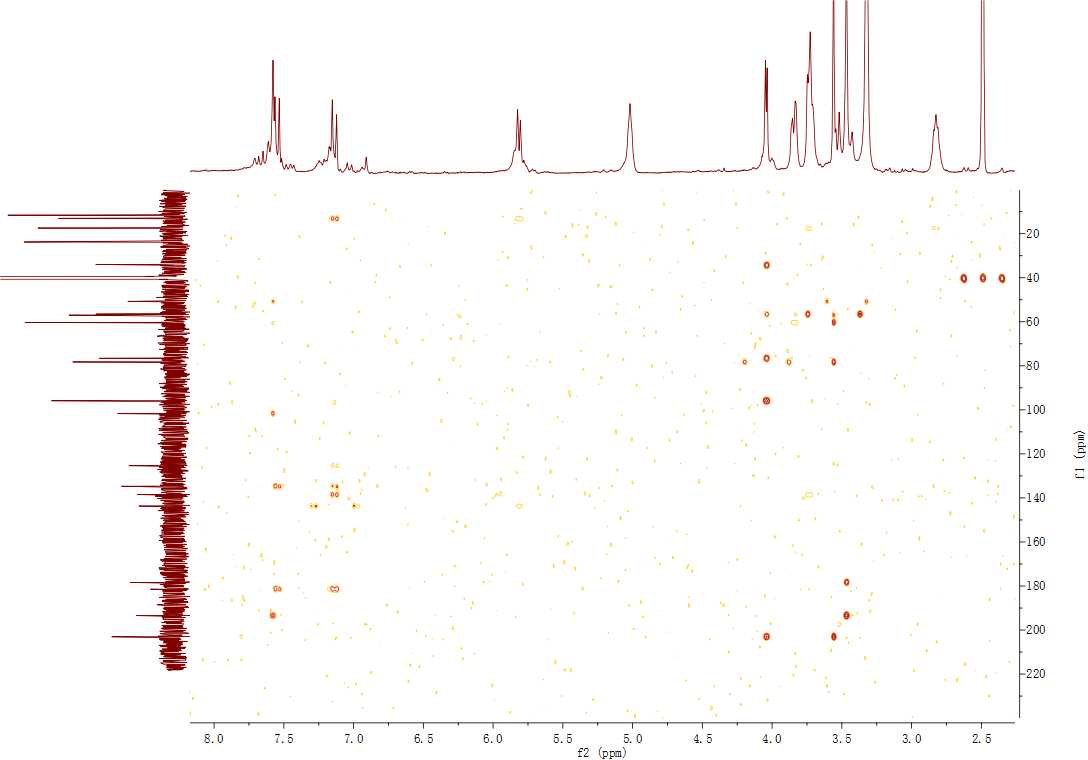


**Figure S25.** The HMBC spectrum of tirandamycin B (**4**) in DMSO-*d*_6_.


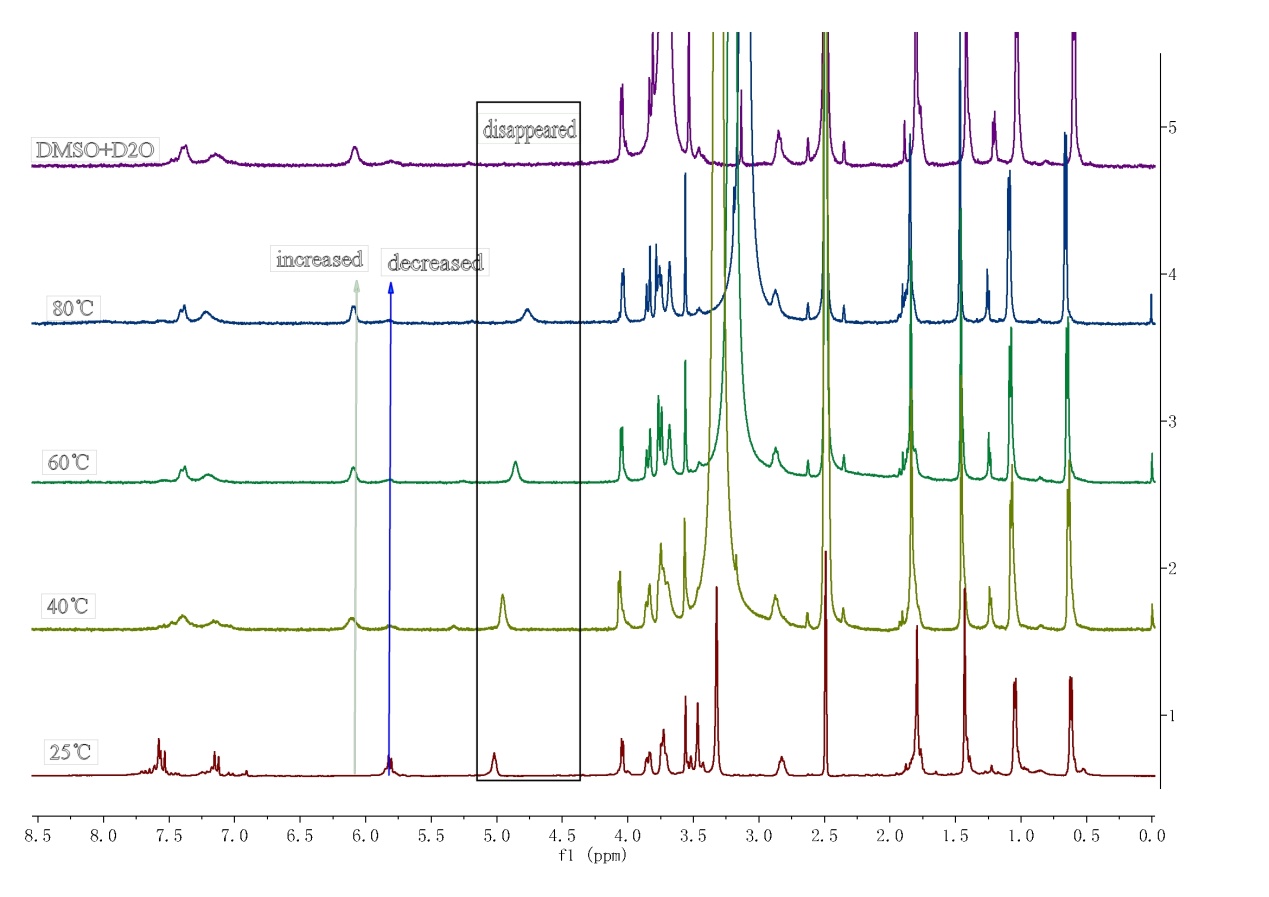


**Figure S26.** The variable temperature experiments and the deuterium exchange experiment for ^1^H NMR of tirandamycin B (**4**).

**Figure S27.** The key HMBC correlations of tirandamycin A (**3**) and tirandamycin B (**4**).


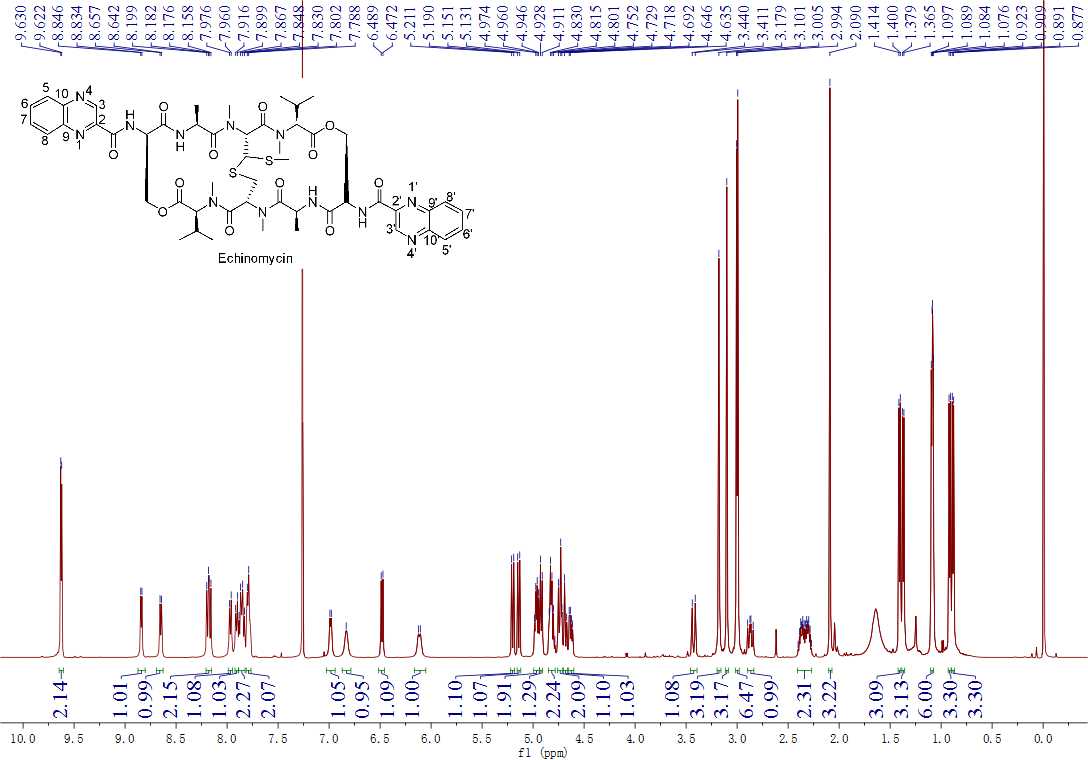


**Figure S28.** The ^1^H NMR spectrum of echinomycin.


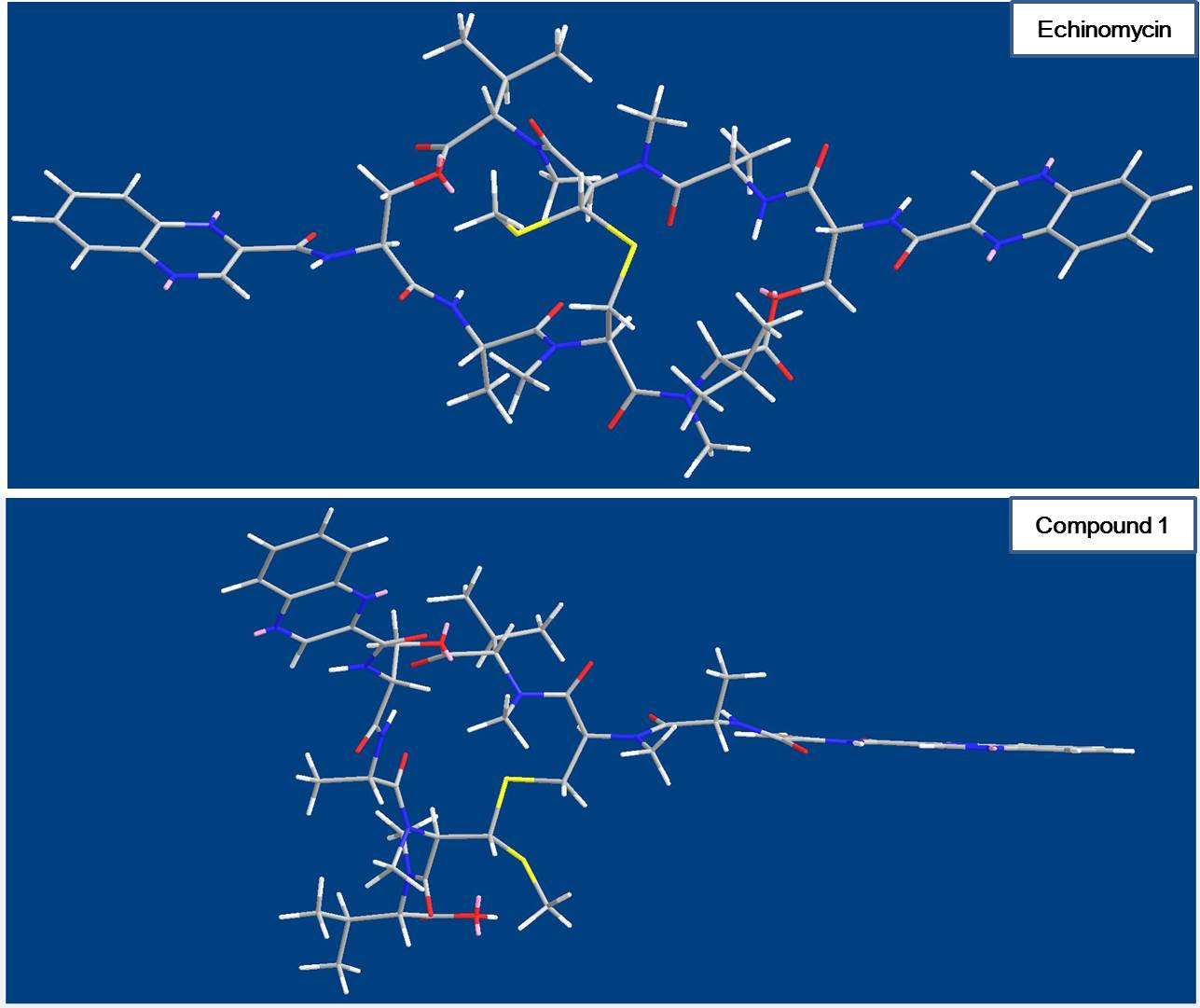


**Figure S29.** The Chem3D MM2 energy minimization models of echinomycin and quinomycin G (**1**).


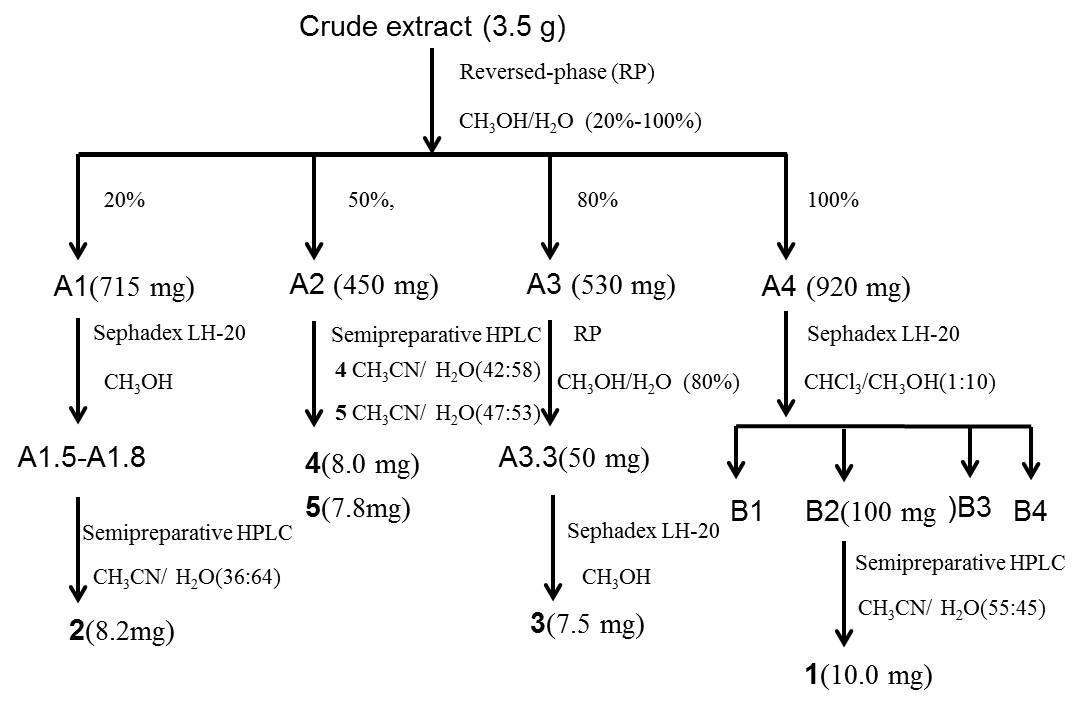


**Figure S30.** The schematic diagram of isolation and identification of compounds **1**–**5**.
